# Modulation of Donor-Acceptor Distance in a Series of Carbazole Push-Pull Dyes; A Spectroscopic and Computational Study

**DOI:** 10.3390/molecules23020421

**Published:** 2018-02-14

**Authors:** Joshua J. Sutton, Jonathan E. Barnsley, Joseph I. Mapley, Pawel Wagner, David L. Officer, Keith C. Gordon

**Affiliations:** 1 Department of Chemistry, University of Otago, P.O. Box 56, Dunedin 9054, New Zealand; sutjo550@otago.ac.nz (J.J.S.); jono.barnsley@postgrad.otago.ac.nz (J.E.B.); mapjo824@student.otago.ac.nz (J.I.M.); 2 ARC Centre of Excellence for Electromaterials Science, University of Wollongong, Wollongong, NSW 2522, Australia; 3 Intelligent Polymer Research Institute/AIIM Faculty, Innovation Campus, University of Wollongong, Wollongong, NSW 2522, Australia

**Keywords:** donor-acceptor dyes, carbazole, cyanoacrylate, thiophene, resonance Raman, TD-DFT, charge transfer

## Abstract

A series of eight carbazole-cyanoacrylate based donor-acceptor dyes were studied. Within the series the influence of modifying the thiophene bridge, linking donor and acceptor and a change in the nature of the acceptor, from acid to ester, was explored. In this joint experimental and computational study we have used electronic absorbance and emission spectroscopies, Raman spectroscopy and computational modeling (density functional theory). From these studies it was found that extending the bridge length allowed the lowest energy transition to be systematically red shifted by 0.12 eV, allowing for limited tuning of the absorption of dyes using this structural motif. Using the aforementioned techniques we demonstrate that this transition is charge transfer in nature. Furthermore, the extent of charge transfer between donor and acceptor decreases with increasing bridge length and the bridge plays a smaller role in electronically mixing with the acceptor as it is extended.

## 1. Introduction

Organic donor acceptor (D-A) dyes are of interest for a wide range of reasons. These mainly stem from their potential use in a range of electronic devices, from OLEDs [1,2,3,4] to photovoltaics [5,6,7,8] to non-linear optics [9,10]. In these fields they often offer advantages over other technologies due to being easier and cheaper to make and being solution processable.

**Figure 1:**
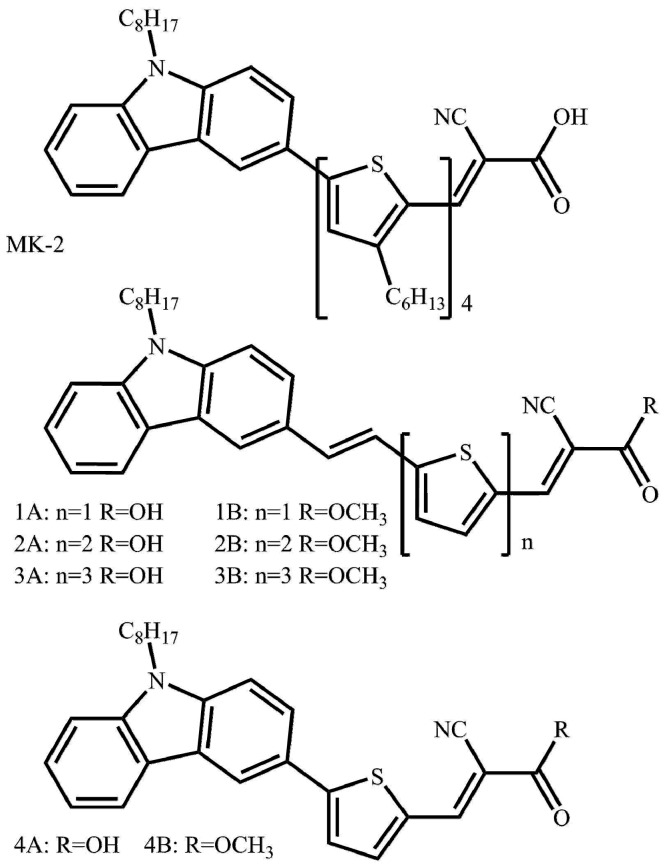
Structure of MK-2, [22] dye (**top**) and the series of D-A dyes studied (**below**).

While a wide range of different donor (carbazole [11,12,13], triphenylamine [14,15,16], dimethylaniline [17,18], fluorene [13] and coumarin [17]) and acceptor (benzothiadiazole [13,15], hexaazatrinaphthalene [16], napthlenediimides [19], dipyridophenazine [20] and cyanoacrylic acid [21]) moieties have been studied this work focuses on the carbazole donor, cyanoacrylic acid acceptor combination. The use of this donor acceptor pairing has been used in a number of studies in the literature [12,22,23,24,25]. It is possible to use this pairing in dye-sensitized solar cells (DSSCs), as has been shown for MK-2 (Figure 1) [22].

This study looks at a series of dyes in which the D-A distance was systematically varied. Further understanding of how the donor acceptor distance influences the optical properties of the dyes is needed. Indeed distance effects have been shown to be important to overall dye performance for DSSCs [14,22] and hydrogen production [26] and a means by which to control charge recombination [27]. A combination of electronic and vibrational spectroscopies and computational techniques is used in an attempt to better understand the influence of the linker group on both the optical and excited state properties for these dyes. The first modification explored was the increase of the bridge from one to three thiophene units (**1A**–**3A** and **1B**-**3B**, Figure 1). The second structural change explored is the effect of increasing the dihedral angle between the donor and bridge (**4A** and **4B**, Figure 1). The dihedral angle of interest is described on in detail in f 2.2. Finally, changes are made to the acceptor, and allow for the comparison of cyanoacrylic acid acceptors with the corresponding methyl ester acceptor. The ester acceptor is explored as it provides a simple method to tune both the electronics and reactivity of the acceptor. The set of compounds with the cyanoacrylic acid acceptor were labeled as set A, while those with the methyl cyanoacrylate acceptor are set B.

An increased linker length is shown to bathochromically shift (0.12 eV (22 nm) terthio vs thio) and decrease the intensity of the lowest energy absorption band, while increasing the dihedral angle resulted in a hypsochromic shift (up to 0.12 eV (20 nm)) in the λ_max_.

## 2. Results and Discussion

### 2.1. Calculated Ground-State Geometries

To assist in understanding the experimental data computational modeling was carried out. The first step was to determine a reliable ground state structure. This was complicated by the fact thiophene chains are subject to rotation around the inter-ring bonds. This phenomenon has a consequence on physical properties [28,29,30]. Single point energies of syn and anti-conformers have been calculated to understand the energetics of this rotation (Table S1). For **2A**/**2B**, the anti-conformer was found to be more stable (by 4.8 kJ mol^−1^ for **2A** and 4.3 kJ mol^−1^ for **2B**) and from the Boltzmann distribution at 293 K, 86% of molecules are expected to occupy the anti-state for **2A**. **3A**/**3B** has several conformers available of which, the anti-anti-conformer was found to be of the lowest energy, 3.9 kJ mol^−1^ lower in energy than the second lowest energy (syn-anti) for **3A** and 3.3 kJ mol^−1^ for **3B** and calculated to account for 80% of the molecular distribution for **3A**. Calculations were performed on **2A**/**2B** as the anti-conformer and **3A**/**3B** as the anti-anti-conformer. In addition it is possible to further validate the calculations (particularly in regard to conformers [28,31]) by comparing the simulated Raman spectra to those observed. The level of correlation may be parametrized by the mean absolute deviations (MADs) in wavenumber for the strongest bands. [15,32,33] A MAD less than 10 cm^−1^ is considered satisfactory and all of the calculated frequencies provide this level of agreement [15,32,33].

**Figure 2:**
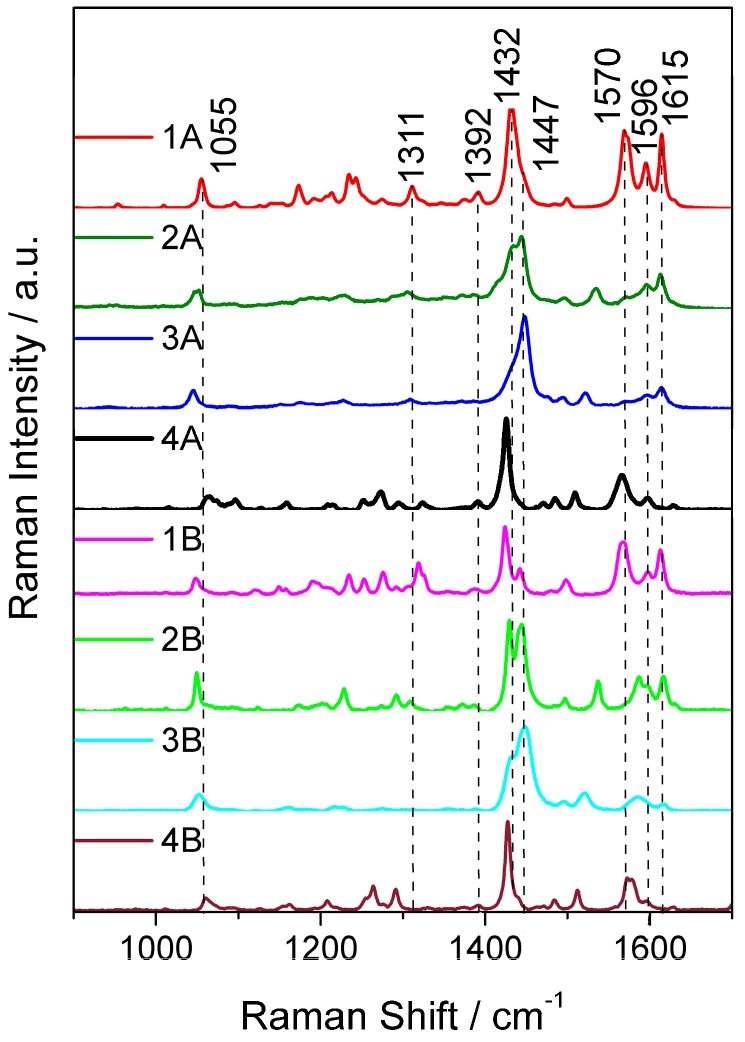
Experimentally measured FT-Raman spectra for the compounds (recorded as solids).

FT-Raman data (Figure 2) shows a number of spectral changes as the thiophene length is increased. The spectral features are dominated by the so-called thiophene B band that lies between 1430 and 1450 cm^−1^. This spectral feature has been studied in other thiophene systems and provides an electronic marker for effective conjugation length [28,34,35,36,37]. The B band results from the in-phase symmetric stretching of the thiophene C=C bonds [38]. Relative to all surrounding bands the thiophene B bands around 1440 cm^−1^ and the 1055 cm^−1^ band, increase in intensity as the bridge length is increased. This results from an increase in polarizability of the thiophene backbone as the bridge length is increased as these modes have backbone/thiophene character. This trend is predicted by the density functional theory (DFT) results. The thiophene B bands also exhibit variation in profile as the thiophene length is altered. In **1A**/**1B** there is a strong band at 1432 cm^−1^ and a shoulder at 1449 cm^−1^, in **2A**/**2B** these peaks are of similar intensity (1433 and 1444 cm^−1^ respectively), while in **3A**/**3B** the 1432 cm^−1^ is decreased to a shoulder on the larger 1448 cm^−1^ peak. These bands are assigned as C–C and C=C symmetric stretches respectively [39,40]. With an increase in conjugation due to a larger number of thiophene units, these bands becomes more intense relative to other peaks as a result of increased polarizability. With substitution from acid to ester the C–C symmetric stretching mode is red shifted with the degree of shift being greatest the shorter dyes (from 1424 to 1432 cm^−1^ for **1A**/**1B**). This results in a greater separation of the C–C and C=C symmetric stretches. DFT calculations predict a single transition to encompass both of these modes and predict a red shift of this single B band as the thiophene backbone is lengthened (−20 cm^−1^ between **3A** and **1A**).

While the thiophene B bands show a gradual shift with the changing length the C=C antisymmetric stretch shows larger changes. The C=C antisymmetric stretch shifts from 1500 cm^−1^ in **1A**/**1B** to 1535 cm^−1^ in **2A**/**2B** and lies at 1522 cm^−1^ in **3A**/**3B**. The intensity of this band follows that of the symmetric C–C stretch for the series of compounds [39].

### 2.2. Electronic Absorption Spectroscopy

**Figure 3:**
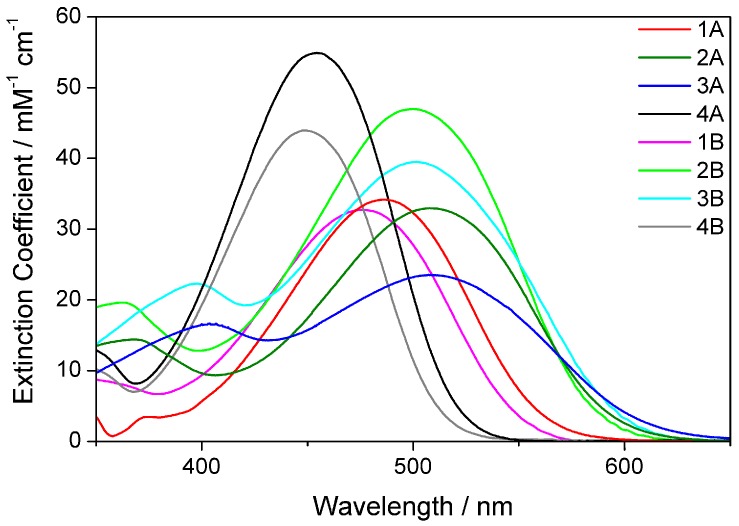
Experimentally collected electronic absorbance spectra for studied compounds, as recorded in DCM.

As the bridge length is increased from mono to terthiophene both the A and B sets show a consistent bathochromic shift in the UV-vis λ_max_ of about 0.12 eV regardless of solvents (Figure 3). There is one exception to this and that is the behavior in dimethylformamide (DMF), and this will be explored later. Both sets show a shift of about 0.11 eV with the addition of one thiophene unit, from mono and bithiophene. However progression to terthiophene resulted in a significantly smaller shift (0.01 eV). The minimal increase in λ_max_ between **2A**/**2B** and **3A**/**3B** could be due to an increase in the twist within the bridge, resulting in a disruption in the degree of conjugation. It should be noted this effect is not predicted by the calculations. When the acceptor is changed from the carboxylic acid unit (set A) to methyl ester (set B) there is a consistent blue shifting of the spectrum.

Further insight into the electronic absorbance of the compounds was gained using time dependent DFT (TD-DFT) calculations. To facilitate a more complete exploration of the electronic structure and optical properties two functionals were used in the calculations; namely B3LYP and CAM-B3LYP. The second of these contains a range correction which has been shown to be effective at modeling charge transfer in systems [15,16,41].

**Figure 4:**
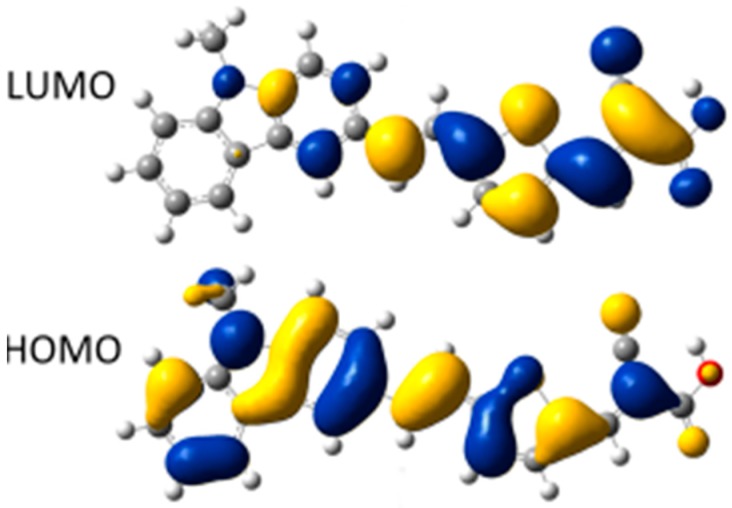
HOMO and LUMO orbital of **1A**, as predicted by TD-DFT calculations (B3LYP/6-31G(d)) (grey = carbon, red = oxygen, blue = nitrogen, yellow = sulfur and white = hydrogen).

The TD-DFT calculations predict the lowest energy peak to be associated with a transition from the highest occupied molecular orbital (HOMO) to lowest unoccupied molecular orbital (LUMO). This transition is predicted to have a net charge transfer from a delocalized orbital to a localized orbital on the linker and acceptor moieties (Figure 4 and Figure S1). Mulliken population analysis calculates a decrease of carbazole electron density upon excitation for this lowest energy transition. This represents charge transfer from carbazole to the acceptor thiophene-cyanoacrylic acid moiety (Table S2). As the bridge length is increased the carbazole donates less electron density (Δ in Table S2) in the charge transfer transition. When the dihedral angle is increased, in **4A** and **4B,** the carbazole charge transfer is greater. This is consistent with electronically isolated nature of the donor and acceptor. When the calculated and experimental λ_max_ of the lowest energy transitions are compared (Table 1 and Table S3, Figure S2) it can be seen that while B3LYP is more accurate for **4A** and **4B**, than CAM-B3LYP which significantly overestimates the energy. When the bridge length is increased (**3A**/**3B**) CAM-B3LYP becomes more accurate. This is consistent with CAM-B3LYP being a range separated hybrid functional, which allows the contribution of exact Hartree-Fock exchange to be modulated over distance. Typically this results in a better prediction of electronic properties [40,42].

**Figure 5:**
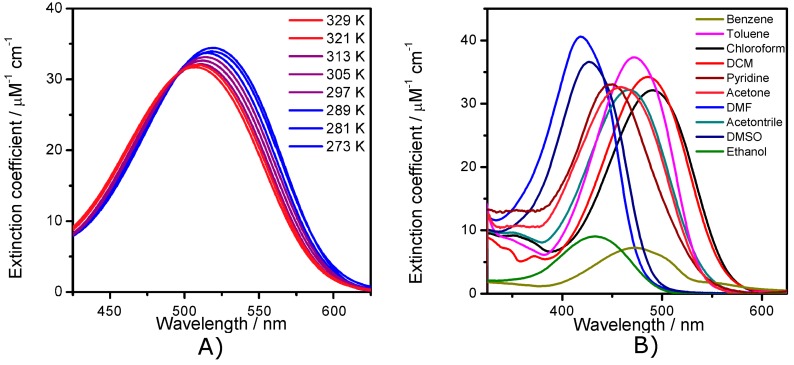
Experimentally measured (**A**) variable temperature absorbance of 1A in CHCl_3_ and (**B**) electronic absorbance of 1A in a range of solvents.

The calculations have demonstrated that the lowest energy transition has significant charge transfer character. Therefore these materials may be expected to interact strongly with perturbation of the environment. Such perturbations may be achieved by changing the solvent (solvatochromism) or altering the temperature (thermochromism) [43]. The effect of temperature on the electronic absorption spectrum of **1A** (typical for that series) is shown in Figure 5A. Blue shifts with increasing temperature are common for charge transfer molecules with large dipole moments. [43,44,45] This can arise due to a decrease of the effective solvent dielectric, [43,46] caused by increasing molecular motion. These results are in agreement with similar thiophene compounds which demonstrate a thermochromic response in the literature [22]. As the temperature of this system is changed there are no sudden changes in intensity or wavelength and no shoulders appear. The smooth experimental spectral response of these materials to temperature and solvent suggest there is no aggregation for this compound under these conditions [47,48].

When the effect of solvent was explored set A compounds showed significant solvatochromism (over 0.43 eV for **1A** between DMF and chloroform (CHCl_3_)) (Table S3), while set B compounds showed a much smaller variation, with a maximum shift of 0.09 eV (for **1B** between acetonitrile (MeCN) and DMF) (Table S3). For set A, the absorbance data displays solvent stabilization of the HOMO energy relative to the LUMO. The degree of stabilization is not strictly relative to solvent polarity, namely Δ*f* (Equation (2)), which suggests the presence of some other solvent-dye interaction as seen in the literature for a triphenylamine equivalent of **1A** [49]. As the effect is not observed in set B it indicates the stabilization is related to interactions between the solvent and acceptor. When this response was studied in more detail increased complexity was observed. Rather than systematic red or blue shifts with the solvent dielectric discontinuous behavior was observed. Toluene, the solvent with the smallest dielectric studied, is in the center of the range with a red shift observed for solvents with an intermediate dielectric (dichloromethane (DCM) and chloroform) and a blue shift for the solvents with the greatest dielectric (MeCN and DMF) (Figure 5B). The solvents that induce the largest λ_max_ blue shifts are strongly polar, can form hydrogen bond and can be slightly basic (ethanol, pyridine, acetone, DMF, MeCN and DMSO). This suggests that either hydrogen bonding involving the carboxylic acid group or deprotonation of that group may be occurring. This phenomenon was explored further using acid and base treatment of **1A** in MeCN and DMF. The addition of acid in MeCN results in no change in the λ_max_, while base treated spectra blue shifts by 0.32 eV (Figure S3). In DMF the reverse is observed, with base treatment resulting in a minimal change in the λ_max_ and the acid treatment red shifting the spectrum by 0.31 eV. This indicates that in MeCN **1A** is fully protonated while in DMF **1A** is nearly fully deprotonated. Since MeCN and DMF are of similar polarity and have similar dielectric constants, the difference in λ_max_ between these solvents (49 nm, 0.31 eV) is thought to be mostly due to deprotonation of the cyanocarboxylate moiety. This result suggests that the large shifts and unusual behavior seen for other basic solvents (ethanol, pyridine, acetone, and DMSO) results from deprotonation events with a remaining contribution from variation of solvent polarity. The sensitivity of these molecules to solvent emphasizes that they have a possible use as environmental probes. In contrast to the large changes observed in the A set there is almost no solvatochromic effect observed in the set B compounds (Figure S2) further supporting the solvatochromism observed in set B is a result of deprotonation, or intramolecular bonding, with the acrylic acid unit. This also shows that by modifying the acceptor unit the dyes sensitivity to solvent polarity can be manipulated. The second lowest energy transition, at around 400 nm shows an insensitivity to the changing solvent, suggesting it is a transition with very little charge transfer character, such as a π-π*.

**Figure 6:**
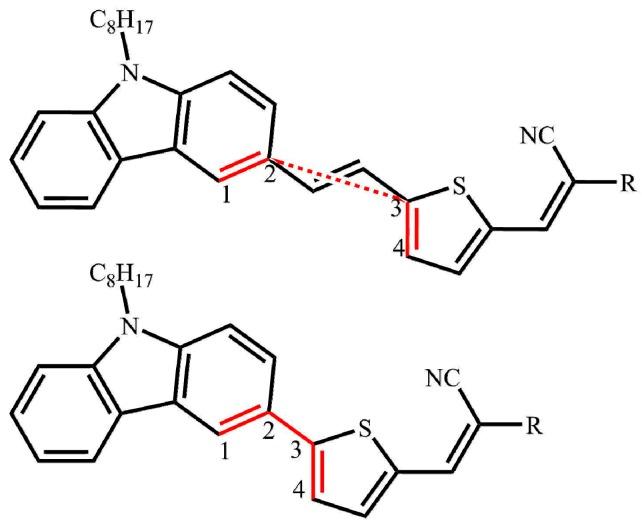
Depiction of dihedral angle between carbazole and thiophene units for **1A**/**1B** and **4A**/**4B**.

For **4A** there is a hypsochromic shift of around 0.06 eV compared to **1A**, while **4B** shows a hypsochromic shift of about 0.12 eV compared to **1B**. This hypsochromic shift may be explained by an increased dihedral angle in **4A**/**4B** which results in a disruption of the conjugation (Figure 6, Table 2). This effect also increases the gap between the HOMO and LUMO.

### 2.3. Resonance Raman Spectroscopy

To further probe the observed electronic transitions and attempt to better understand how the implemented structural changes result in the observed optical changes, resonance Raman spectroscopy (RRS) was carried out at a number of excitation wavelengths across the absorption bands of the compounds. Resonance Raman spectroscopy shows band enhancements for modes that are associated with the resonant chromophore [16,20,41,50,51], thus it is possible to provide experimental evidence for the nature of the observed chromophores to support the calculated electronic transitions data.

By scanning the absorption profile, and monitoring the resonantly enhanced vibrations, the nature of the electronic transitions occurring can be probed. In the context of this study charge transfer excitation could lead to the enhancement of either donor or acceptor modes, enhancement of bridging modes would be consistent with the bridge playing a role in this transition [50,52,53,54,55].

**Figure 7:**
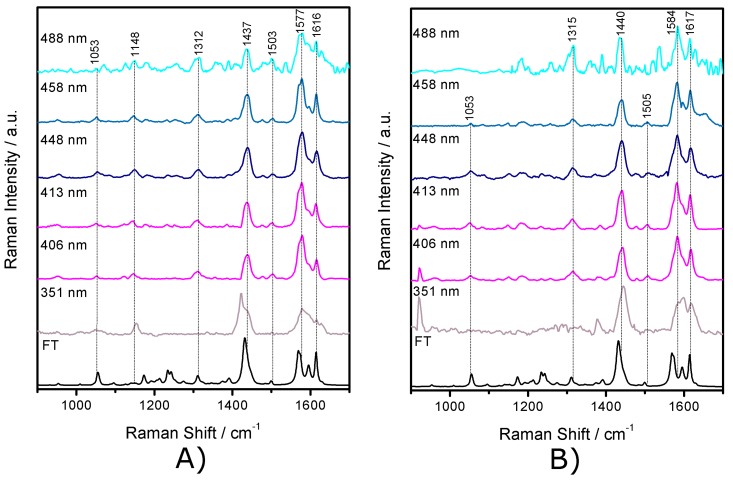
Experimental resonance Raman spectra for **1A** (1 mM) recorded in (**A**) toluene and (**B**) MeCN at the wavelengths listed.

**Figure 8:**
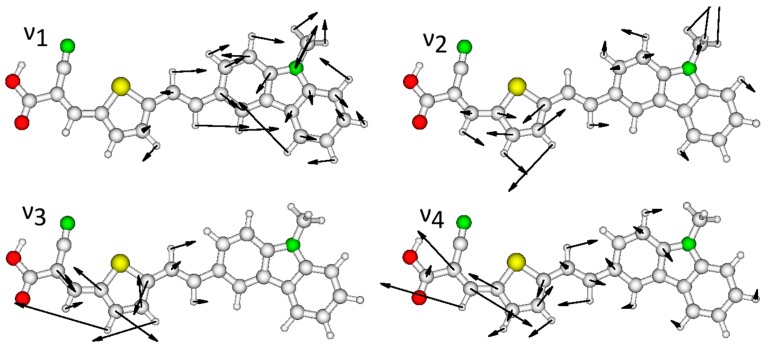
Key vibrational modes of **1A**, as modeled at the B3LYP/6-31G(d) level (grey = carbon, red = oxygen, green = nitrogen, yellow = sulfur and white = hydrogen).

In the resonance Raman data (Figure 7 and Figures S8–S14) excitation at 351 nm results in the strong enhancement of a mode at 1437 cm^−1^. This mode is linker based (ν_2_, Figure 8), suggesting the higher energy transition, observed at around 350 nm in the electronic absorbance spectra is centered on the linker. As the excitation energy is moved to lower energy (406–488 nm) increased enhancement of vibrational modes at 1312, 1503 and 1577 cm^−1^ is observed (ν_1_, ν_3_ and ν_4_ respectively). These modes are located primarily on either the donor (ν_1_) or acceptor (ν_3_ and ν_4_) units. The resonant enhancement of such vibrations suggests changes in electron density in these regions and provides experimental evidence for charge transfer behavior for the lowest energy optical absorption. Relative enhancement of vibrations ν_1_, ν_3_ and ν_4_ compared to the thiophene ν_2_ band suggests that electron density change on the thiophene backbone is less drastic than at either the carbazole or cyano-carboxylate. This is supported by the TD-DFT calculations. These predict that the thiophene units have electron density changes of less than 50% of that seen on the donor or acceptor (Table S2). There are blue shifts in most bands with increases in polarity of the solvent (toluene to DCM to MeCN); this results from the increased stabilization of the ground state. The RRS data indicates that regardless of the bridge length the nature of the lowest energy transition remains relatively constant with most of the change in structure and electron density occurring on the donor and acceptor units.

### 2.4. Emission Spectroscopy

**Figure 9:**
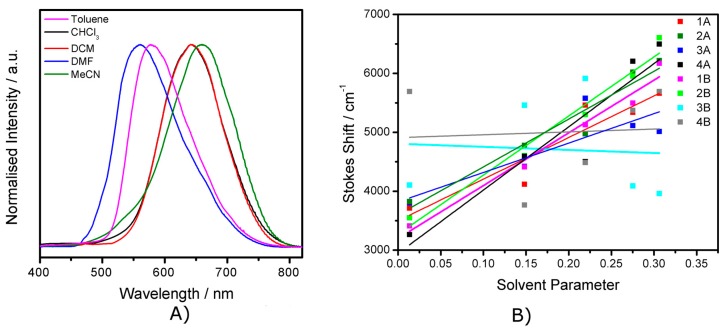
(**A**) Emission spectra for **1A** measured in a range of solvents and (**B**) Lippert-Mataga plots for all the compounds based on experimental absorption and emission data.

In addition to the ground state changes the effect of the structural modifications on the excited stated was also examined. Understanding how the excited state is influenced by the structural changes is important as most the potential uses for D-A compounds exploit the excited state. All of the compounds show considerable solvatochromism in their emission spectra (Table 3, Figure 9A and Figure S4), with **1A** showing the greatest change. **1A** varies by 0.33 eV (98 nm) with the highest energy emission occurring at 561 nm in DMF and the lowest energy in MeCN at 659 nm. A bathochromic shift with respect to solvent polarity is associated with a more polar excited state, consistent with a charge transfer transition, as indicated by the RRS and calculations.

In order to better understand the solvochromatic response Lippert-Mataga analysis was carried out. This analysis links the Stokes shift (${\tilde{\nu }}_{A}-{\tilde{\nu }}_{B})$ to some of the solvents properties (Equation (1)) and provides an experimental way to estimate the change in dipole upon excitation [44,56,57,58]:

(1) $${\tilde{\nu }}_{A}-{\tilde{\nu }}_{B}=\frac{2{\left(\mu_{e}-\mu_{g}\right)}^{2}}{4\pi \varepsilon_{0}}\frac{\Delta f}{hca^{3}}+C$$

where ${\tilde{\nu }}_{A}$ and ${\tilde{\nu }}_{B}$(cm^−1^) are the experimentally determined lowest energy absorbance and emission maximum, *h* (J s), $\varepsilon_{0}$ (J^−1^ C^2^ m^−1^) and *c* (cm s^−1^) are constants, $\mu_{e}$ and $\mu_{g}$ (D) is the dipole moment in the ground and excited states respectively, a (m) is the Onsager radius and Δf is the solvent polarity parameter. Δf can be determined using the dielectric constant (ε) and refractive index (n) of the solvent, via Equation (2):

(2) $$\Delta f=\left(\frac{\varepsilon -1}{2\varepsilon +1}\right)-\left(\frac{n^{2}-1}{2n^{2}-1}\right)$$

With the exception of **3A**/**3B** (vide infra) the compounds were found to have a linear response for Stokes shift with Δ*f*. This corresponds to a change in dipole (Δ*μ*, *µ_e_* − *µ_g_*) of 10–15 Debye (Table S4, Figure 9B) [44]. This suggests modest charge transfer in the compounds. Between **1A**/**1B** and **2A**/**2B** an increase was seen in Δ*μ*. This may be due to the increased donor-acceptor distance which increases the degree of charge separation and therefore dipole moment, in the excited state. The Onsager radius was determined using the Gaussian Volume keyword. The Lippert Mataga plots showed a deviation from the linear trend for **3A**/**3B** with the most polar solvents, DMF and MeCN showing a decrease in Stokes shift. This suggests that the longer bridge is susceptible to direct solvent interactions.

Variable temperature emission experiments were carried out on **1A** to **3A** in a variety of solvents and have been included in the Supplementary Materials (Figures S5–S7). These show a consistent increase in emission intensity and slight red shift (~0.02 eV) as the temperature is decreased. As discussed earlier, the red shift results from a reduction in solvent molecular motion, thus a more effective stabilization of the exited state [44].

The quantum yield for **1B** to **4B** were recorded in toluene and DCM (Table 4). While **1B**, **2B** and **4B** show a trend, with a decreasing quantum yield with decreased chain length. **1B**, **2B** and **4B** show a significant drop in quantum yield between toluene and DCM, while **3B** is unchanged. When the decay rates in DCM are calculated (Table 4, Equation (S1)) it is observed that the radiative decay (*k*_r_) shows a variation for **1B**, **2B** and **3B** by a factor of 2, with a decrease as the bridge length is increased. The non-radiative decay rate (*k*_nr_) however, shows minimal change between **1B** and **3B**, but an order of magnitude decrease for **2B**. This means that the bithiophene bridge inhibits non-radiative decay pathways.

**Figure 10:**
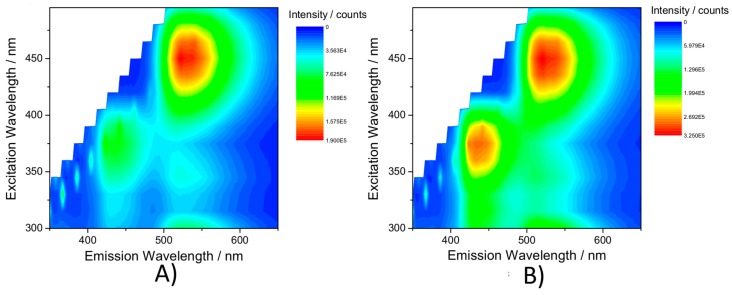
Excitation vs emission plots for (**A**) **4A** and (**B**) **4B** recorded in toluene.

Increasing the dihedral angle between donor and acceptor (**4B**) shows a significant perturbation of the excited state. **4A**/**4B** show an unusual dual emission behavior (Figure 10). Strong emission at 525 nm is observed when exciting with 450 nm and a higher energy emission at 425 nm when exciting at 375 nm. The higher energy emission was not observed in **1A**–**3A**/**1B**–**3B**. This higher energy emission suggests that the 375 nm transition in **4A**/**4B** populates an excited state energetically isolated from the lower energy charge transfer emission. The presence of this state in **4A**/**4B** suggests it is linked to the increased angle between the carbazole donor and acceptor. It is plausible this is due to a π to π* state.

### 2.5. The Effect of the Bridge on Properties

The series show a number of effects on optical and excited state properties as a function of the bridge between the donor and acceptor groups. Firstly, the elongation of the bridge for both sets of compounds (**A** and **B**, compounds **1** through **3**) show a red shift in the charge transfer transition, but this effect is greatest between the mono and bithiophene with a muted effect, experimentally, on going from bi- to terthiophene (Table 1). This experimental finding is not borne out by calculations which show a continuous red shift from **1** to **3** (Table 1). The calculations do show that the electron density of the HOMO and LUMO (the orbitals associated with the charge transfer transition) increases on the bridge with elongation (Table S2). The calculations also succeed in predicting a lower intensity for the charge transfer band on going from **2** to **3** (Figure S2). Secondly, compounds **1A**, **1B**, **2A**, **2B** show a linear response for Stokes shift with solvent parameter (Figure 9B). The terthiophene bridged system however does not respond in this fashion and this suggests specific solvent interaction that may also explain the optical transition properties. It is also notable that **2B** has the longest excited state lifetime and highest quantum yield despite having a lower energy than **1B**. The shorter lifetime and lower quantum yield for **3B** supports the suggestion, from other data, that this compound, and by inference the terthiophene bridge in general, has conformational flexibility that is deactivating the excited state. This suggests that for these types of donor-acceptor the bithiophene is the optimal bridge if longer lifetimes are required.

## 3. Materials and Methods

### 3.1. Synthesis

**Scheme 1:**
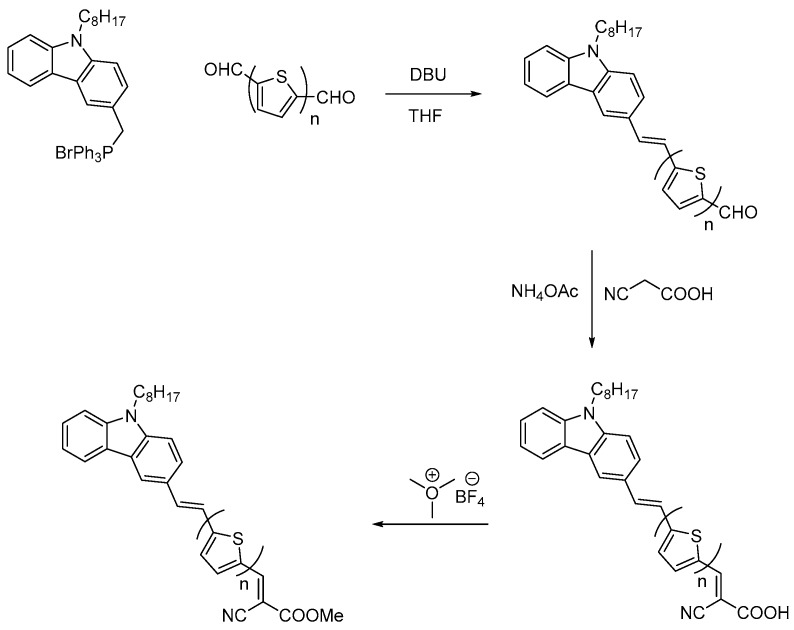
Reaction scheme for synthesis of **1A**–**3A** and **1B**–**3B**.

**Scheme 2:**
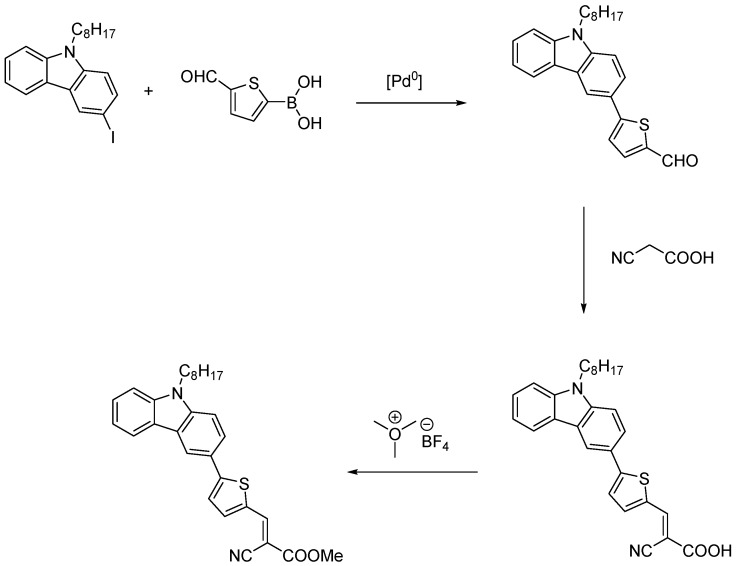
Reaction scheme for **4A**/**4B**.

Triphenylmethyl(9-octylcarbazol-3-yl)phosphonium bromide and 3-iodo-9-octylcarbazole were synthesized according to procedures described before [59,60]. The other compounds were obtained commercially and used without further purification unless stated differently. The synthetic procedures were not optimized. The synthesis process for **1A** to **3A** and **1B** to **3B**) is outlined in Scheme 1, while the process for **4A** and **4B** is outlined in Scheme 2. NMR spectra were recorded on an Avance 400 spectrometer (Bruker, Billerica, MA, USA). The following abbreviations were used: s = singlet, d = doublet, dd = doublets of doublets, ddd = doublets of doublets of doublets, m = multiplet, t = triplet. All coupling constants *J* are expressed in hertz (Hz). Chemical shifts are given in parts-per-million (ppm). Tetramethylsilane was used as the internal reference. Mass spectra were recorded on a Polaris Q (ThermoFisher, Waltham, MA, USA) or Hewlett Packard 5973 (Agilent, Santa Clara, CA, USA) instrument.

#### 3.1.1. General Procedure for Wittig Condensation

Dialdehyde (1 mmol) and triphenylmethyl(9-ethylcarbazol-3-yl)phosphonium bromide (620 mg, 0.98 mmol) were dissolved in dry THF (10 mL) then 1,8-diazabicyclo[5.4.0]undec-7-ene (DBU) (609 mg, 4.0 mmol) was added. The resulting mixture was stirred at room temperature for 90 min then the solvent was removed under vacuum at 50 °C. The product was isolated on chromatographic column using CH_2_Cl_2_ as an eluent. To obtain pure *E* isomer, the product was dissolved in CH_2_Cl_2_ (10 mL) then trifluoroacetic acid (5 mL) and water (3 mL) was added. The resulting mixture was stirred at room temperature for 20 min, then neutralized with concentrated ammonia. CH_2_Cl_2_ (50 mL) was added to the mixture, the organic phase was separated, dried over magnesium sulphate and evaporated to dryness. The crude product was purified on silica using dichloromethane as an eluent.

*(E)-5-[2-(9-Octylcarbazol-3-yl)ethenyl]thiophene-2-carbaldehyde* (**1**). Yield: 57%; ^1^H-NMR (400 MHz, CDCl_3_) δ: 9.85 (s, 1H, CHO), 8.22 (dd, 1H, *J* = 1.6 and 0.4 Hz, Carb-H4), 8.11 (ddd, 1H, *J* = 8.2, 1.2 and 0.8 Hz, Carb-H5), 7.66 (d, 1H, *J* = 4.0 Hz, Th-H3), 7.64 (dd, 1H, *J* = 8.6 and 0.4 Hz, Carb-H2), 7.48 (ddd, 1H, *J* = 8.2, 7.2 and 1.2 Hz, Carb-H6), 7.42–7.35 (m, 3H, vinyl-H + Carb-H1), 7.28–7.24 (m, 2H, Carb-H7, H8), 7.14 (d, 1H, *J* = 4.0 Hz, Th-H4), 4.29 (t, 2H, *J* = 7.6 Hz, N-CH_2_), 1.93–1.83 (m, 2H, CH_2_), 1.45–1.18 (m, 10H, 5 × CH_2_), 0.86 (t, 3H, *J* = 7.1 Hz, CH_3_); ^13^C-NMR (100 MHz, CDCl_3_) δ: 182.4, 153.7, 141.0, 140.9, 140.8, 137.4, 134.4, 127.0, 126.1, 125.5, 123.4, 122.8, 120.5, 119.5, 199.4, 118.1, 109.1, 109.0, 43.3, 31.8, 29.4, 29.2, 29.0, 27.3, 22.6, 14.1; HRMS (ESI, [M + H]^+^): found: 416.2055, requires for C_27_H_30_NOS: 416.2043.

*(E)-5′-[2-(9-Octyl-9H-carbazol-3-yl)ethenyl]-(2,2′-bithiophene)-5-carbaldehyde* (**2**). Yield: 67%; ^1^H-NMR (400 MHz, CDCl_3_) δ: 9.85 (s, 1H, CHO), 8.18 (dd, 1H, *J* = 1.6 and 0.4 Hz, Carb-H4), 8.11 (ddd, 1H, *J* = 8.2, 1.2 and 0.8 Hz, Carb-H5), 7.66 (d, 1H, *J* = 4.0 Hz, Th-H), 7.62 (dd, 1H, *J* = 8.6 and 0.4 Hz, Carb-H2), 7.47 (ddd, 1H, *J* = 8.2, 7.2 and 1.2 Hz, Carb-H6), 7.42–7.35 (m, 3H, Vinyl-H + Carb-H1), 7.28–7.17 (m, 4H, Carb-H7, H8, 2 × Th-H), 7.00 (d, 1H, *J* = 4.0 Hz, Th-H), 4.28 (t, 2H, *J* = 7.7 Hz, N-CH_2_), 1.93–1.82 (m, 2H, CH_2_), 1.43–1.18 (m, 10H, 5 × CH_2_), 0.86 (t, 3H, *J* = 7.1 Hz, CH_3_); ^13^C-NMR (100 MHz, CDCl_3_) δ: 182.3, 147.5, 145.9, 140.9, 140.5, 137.4, 133.4, 131.3, 127.6, 126.9, 126.3, 126.0, 124.4, 123.8, 122.8, 120.4, 119.2, 118.9, 118.4, 109.1, 109.0, 43.3, 31.8, 29.4, 29.2, 29.0, 27.3, 22.6, 14.0; HRMS (ESI, [M + H]^+^): found: 498.1931, requires for C_31_H_32_NOS_2_: 498.1920.

*(E)-5′-[2-(9-Octylcarbazol-3-yl)ethenyl]-(2,2′;5′,5″-terthiophene)-5-carbaldehyde* (**3**). Yield: 67%; ^1^H-NMR (400 MHz, CDCl_3_) δ: 9.86 (s, 1H, CHO), 8.19 (dd, 1H, *J* = 1.7 and 0.4 Hz, Carb-H4), 8.11 (ddd, 1H, *J* = 8.6, 1.1 and 0.7 Hz, Carb-H5), 7.67 (d, 1H, *J* = 4.0 Hz, Th-H), 7.64 (dd, 1H, *J* = 8.8 and 0.4 Hz, Carb-H2), 7.48 (ddd, 1H, *J* = 8.4, 7.1, 1.2 Hz, Carb-H6), 7.42–7.36 (m, 2H, Vinyl-H + Carb-H1), 7.29 (d, 1H, *J* = 3.7 Hz, Th-H), 7.28–7.12 (m, 6H, Carb-H7, H8, 3 × Th-H), 6.99 (d, 1H, *J* = 4.0 Hz, Th-H), 4.30 (t, 2H, *J* = 7.4 Hz, N-CH_2_), 1.92–1.82 (m, 2H, CH_2_), 1.44–1.18 (m, 10H, 5 × CH_2_), 0.86 (t, 3H, *J* = 7.1 Hz, CH_3_); HRMS (ESI, [M + H]^+^): found: 580.1822, requires for C_35_H_34_NOS_3_: 580.1797.

#### 3.1.2. General Procedure for the Knoevenagel Condensation

Aldehyde (0.4 mmol), cyanoacetic acid (350 mg, 4.1 mmol) and ammonium acetate (309 mg, 4.0 mmol) were dissolved in a mixture of tetrahydrofuran (6 mL) and glacial acetic acid (6 mL). The resulting mixture was stirred at 70°C for 3 h then quenched with water (50 mL). The resulting solid was filtered off, washed several times with water then vacuum dried. Only one isomer was obtained.

*(E)-2-Cyano-3-{5-[2-(9-octyl-9H-carbazol-3-yl)ethenyl]thiophen-2-yl}acrylic acid* (**1A**). Yield: 99%; ^1^H-NMR (400 MHz, DMSO-*d*_6_) δ: 8.50 (d, 1H, *J* = 1.3 Hz, Carb-H4), 8.46 (s, 1H, vinyl CN-H), 8.17 (ddd, 1H, *J* = 8.2, 0.8 and 0.4 Hz, Carb-H6), 7.95 (d, 1H, *J* = 4.0 Hz, Th-H3), 7.81 (dd, 1H, *J* = 8.8, 1.8 Hz, Carb-H5), 7.65–7.54 (m, 3H, vinyl-H + Carb-H1), 7.50–7.40 (m, 2H, Carb-H2, H8, Th-H4), 7.30 (ddd, 1H, *J* = 7.8, 7.7 and 0.7 Hz, Carb-H7), 4.39 (t, 2H, *J* = 7.1 Hz, N-CH_2_), 1.82–1.71 (m, 2H, CH_2_), 1.34–1.12 (m, 10H, 5 × CH_2_), 0.81 (t, 3H, *J* = 7.1 Hz, CH_3_); HRMS (ESI, [M − H]^−^): found: 481.1937, requires for C_30_H_29_N_2_O_2_S: 481.1955.

*(E)-2-Cyano-3-{5-[2-(9-octyl-9H-carbazol-3-yl)ethenyl]-(2,2′-bithiophen-2-yl)}acrylic acid* (**2A**). Yield: 99%; ^1^H-NMR (400 MHz, DMSO-*d*_6_) δ: 8.40 (d, 1H, *J* = 1.5 Hz, Carb-H4), 8.17 (ddd, 1H, *J* = 8.1, 0.8 and 0.5 Hz, Carb-H6), 8.14 (s, 1H, vinyl CN-H), 7.73 (d, 1H, *J* = 4.0 Hz, Th-H), 7.72 (dd, 1H, *J* = 8.9 and 1.7 Hz, Carb-H5), 7.61–7.57 (m, 2H, Th-H), 7.50–7.44 (m, 4H, Carb-H2, H8, vinyl-H), 7.26–7.19 (m, 3H, Carb-H2, H7, Th-H), 4.38 (t, 2H, *J* = 7.1 Hz, N-CH_2_), 1.83–1.71 (m, 2H, CH_2_), 1.35–1.12 (m, 10H, 5 × CH_2_), 0.81 (t, 3H, *J* = 7.1 Hz, CH_3_); HRMS (ESI, [M-H]^−^): found: 563.1818, requires for C_34_H_31_N_2_O_2_S_2_: 563.1832.

*(E)-2-Cyano-3-{5-[2-(9-octyl-9H-carbazol-3-yl)ethenyl]-(2,2′;5′,5″-terthiophen-2-yl)}acrylic acid* (**3A**). Yield: 99%; ^1^H-NMR (400 MHz, DMSO-*d*_6_) δ: 8.39 (d, 1H, *J* = 1.5 Hz, Carb-H4), 8.17 (ddd, 1H, *J* = 8.1, 7.8, 0.5 Hz, Carb-H6), 8.11 (s, 1H, vinyl CN-H), 7.74–7.70 (m, 2H, Carb-H5, Th-H), 7.61–7.58 (m, 2H, Carb-H1, Th-H), 7.50–7.43 (m, 4H, Carb-H2, H8, vinyl-H), 7.37 (d, 1H, *J* = 3.7 Hz, Th-H), 7.36 (d, 1H, *J* = 3.7 Hz, Th-H), 7.22 (ddd, 1H, *J* = 8.0, 7.8 and 0.7 Hz, Carb-H7), 7.19–7.13 (m, 2H, Th-H), 4.39 (t, 2H, *J* = 7.1 Hz, N-CH_2_), 1.83–1.73 (m, 2H, CH_2_), 1.35–1.12 (m, 10H, CH_2_), 0.82 (t, 3H, *J* = 7.1 Hz, CH_3_); HRMS (ESI, [M + H]^+^): found: 647.1872, requires for C_38_H_35_N_2_O_2_S_3_: 647.1855.

#### 3.1.3. General Procedure for Esterification of Cyanoacrylic Acids:

Acid (0.047 mmol) was suspended in dry Et_2_O (30 mL), ethyldiisopropyl amine (0.2 mL) was added followed by trimethyloxonium tetrafluoroborate (0.19 mmol). The resulting slurry was stirred at room temperature for 3 h then quenched with methanol. The solvents were removed under vacuum and the remaining was filtered through pad of silica using CH_2_Cl_2_ as an eluent.

*Methyl (E)-2-CYANO-3-{5[2-(9-octyl-9H-carbazol-3-yl)ethenyl]thiophen-2-yl}acrylate* (**1B**). Yield: 99%; ^1^H-NMR (400 MHz, CDCl_3_) δ: 8.26 (d, 1H, *J* = 0.5 Hz, Carb-H4), 8.23 (dd, 1H, *J* = 8.2 and 7.8 Hz, Carb-C6), 8.11 (ddd, 1H, *J* = 7.8, 1.1 and 0.8 Hz, Carb-H5), 7.66–7.62 (m, 2H, Carb-H7, Th-H), 7.50–7.48 (m, 3H, Carb-H2, H8, vinyl-H), 7.42–7.37 (m, 3H, Carb-H1, vinyl-H, vinyl CN-H), 7.13 (d, 1H, *J* = 3.9 Hz, Th-H), 4.29 (t, 2H, *J* = 7.1 Hz, N-CH_2_), 3.91 (s, 3H, COOCH_3_), 1.92–1.83 (m, 2H, CH_2_), 1.43–1.20 (m, 10H, CH_2_), 0.86 (t, 3H, *J* = 7.1 Hz, CH_3_); HRMS (ESI, (M + H)^+^): found: 497.2271, requires for C_31_H_33_N_2_O_2_S: 497.2257.

*Methyl (E)-2-Cyano-3-{5-[2-(9-octyl-9H-carbazol-3-yl)ethenyl]-(2,2′-bithiophen-2-yl)}acrylate* (**2B**). Yield: 99%; ^1^H-NMR (400 MHz, CDCl_3_) δ: 8.24 (d, 1H, *J* = 0.5 Hz, Carb-H4), 8.19 (dd, 1H, *J* = 8.1 and 7.8 Hz, Carb-C6), 8.11 (ddd, 1H, *J* = 7.8, 1.1 and 0.7 Hz, Carb-H5), 7.66 (dd, 1H, *J* = 7.4 and 0.6 Hz, Carb-H2), 7.62 (dd, 1H, *J* = 8.1 and 1.6 Hz, Carb-H8), 7.47 (ddd, 1H, *J* = 8.1, 7.0 and 1.4 Hz, Carb-H7), 7.42–7.36 (m, 2H, vinyl-H), 7.31 (d, 1H, *J* = 4.0 Hz, Th-H), 7.27–7.25 (m, 2H, Carb-H1, vinyl CN-H), 7.24 (d, 1H, *J* = 4.0 Hz, Th-H), 7.19 (d, 1H, *J* = 4.0 Hz, Th-H), 7.02 (d, 1H, *J* = 4.0 Hz, Th-H), 4.29 (t, 2H, *J* = 7.1 Hz, N-CH_2_), 3.91 (s, 3H, COOCH_3_), 1.93–1.83 (m, 2H, CH_2_), 1.45–1.17 (m, 10H, 5 × CH_2_), 0.87 (t, 3H, *J* = 7.1 Hz, CH_3_); HRMS (ESI, [M + H]^+^): found: 579.2150, requires for C_35_H_35_N_2_O_2_S_2_: 579.2134.

*Methyl (E)-2-Cyano-3-{5-[2-(9-octyl-9H-carbazol-3-yl)ethenyl]-(2,2′;5′,5″-terthiophen-2-yl)}acrylate* (**3B**). Yield: 99%; ^1^H-NMR (400 MHz, CDCl_3_) δ: 8.21 (d, 1H, *J* = 0.7 Hz, Carb-H4), 8.16 (dd, 1H, *J* = 8.1 and 7.8 Hz, Carb-C6), 8.10 (ddd, 1H, *J* = 7.8, 1.1 and 0.7 Hz, Carb-H5), 7.62 (dd, 1H, *J* = 7.4 and 0.7 Hz, Carb-H2), 7.62 (dd, 1H, *J* = 8.6 and 1.7 Hz, Carb-H8), 7.47 (ddd, 1H, *J* = 8.6, 7.1 and 1.7 Hz, Carb-H7), 7.40–7.34 (m, 2H, vinyl-H), 7.29 (d, 1H, *J* = 4.0 Hz, Th-H), 7.27–7.16 (m, 4H, Carb-H1, vinyl CN-H, 2 × Th-H), 6.96 (d, 1H, *J* = 4.0 Hz, Th-H); 4.28 (t, 2H, *J* = 7.1 Hz, N-CH_2_), 3.89 (s, 3H, COOCH_3_), 1.93–1.81 (m, 2H, CH_2_), 1.44–1.16 (m, 10H, 5 × CH_2_), 0.86 (t, 3H, *J* = 7.1 Hz, CH_3_); HRMS (ESI, [M + H]^+^): found: 661.2022, requires for C_39_H_37_N_2_O_2_S_3_: 661.2012.

*5-(9-Octylcarbazol-3-yl)thiophene-2-carbaldehyde* (**4**). 5-Formyl-2-thienylboronic acid (1.550 g, 10 mmol) and 3-iodo-9-octylcarbazole (2.700 g, 6.7 mmol) were dissolved in tetrahydrofuran (50 mL). The solution was degassed by argon for 15 minutes then was mixed with degassed aqueous solution of potassium carbonate (20 mL, 1 mol) and tetrakis(triphenylphosphin)palladium(0) (Pd(PPh_3_)_4_) (420 mg, 4% mol). The resulting mixture was stirred at reflux for 4 h then cooled down. The solvents were removed under vacuum at 50 °C and the remaining was purified on silica using dichloromethane: hexane mixture (4:1) as an eluent. The first bright yellow fraction was collected and recrystallized from methanol to give the product as yellow needles. Yield: 28%; ^1^H-NMR (400 MHz, CDCl_3_) δ: 9.88 (s, 1H, CHO), 8.39 (dd, 1H, *J* = 1.9 and 0.5 Hz, Carb-H4), 8.13 (ddd, 1H, *J* = 8.0, 1.2 and 0.7 Hz, Carb-H5), 7.79–7.74 (m, 2H, Carb-H2, Th-H3), 7.50 (ddd, 1H, *J* = 8.2, 7.1 and 1.2 Hz, Carb-H7), 7.45–7.40 (m, 3H, Carb-H1, H8, Th-H4), 7.27 (ddd, 1H, *J* = 8.0, 7.1 and 1.0 Hz, Carb-H6), 4.31 (t, 2H, *J* = 7.1 Hz, N-CH_2_), 1.93–1.83 (m, 2H, CH_2_), 1.43–1.19 (m, 10H, 5 × CH_2_), 0.86 (t, 3H, *J* = 7.1 Hz CH_3_); ^13^C-NMR (100 MHz, CDCl_3_) δ: 182.9, 156.4, 141.3, 141.1, 137.8, 136.2, 135.1, 128.3, 126.4, 124.4, 124.1, 123.5, 122.9, 120.6, 119.6, 118.6, 109.3, 43.3, 31.8, 29.3, 29.2, 29.0, 27.3, 22.6, 14.1; HRMS (ESI, [M + H]^+^): found: 391.1980, requires for C_25_H_29_NOS: 391.1964.

*2-Cyano-3-[5-(9-octylcarbazol-3-yl)thiophen-2-yl]acrylic acid* (**4A**). This compound was synthesized according to the Knoevenagel condensation as described above. Yield: 99%; ^1^H-NMR (400 MHz, DMSO-*d*_6_) δ: 8.64 (d, 1H, *J* = 1.5 Hz, Carb-H4), 8.64 (s, 1H, vinyl CN-H), 8.30 (dd, 1H, *J* = 8.0 and 0.8 Hz, Carb-H5), 8.04 d (1H, *J* = 4.1 Hz, Th-H3), 7.88 (dd, 1H, *J* = 8.7 and 1.5 Hz, Carb-H2), 7.80 (d, 1H, *J* = 4.1 Hz, Th-H4), 7.71 (d, 1H, *J* = 8.7 Hz, Carb-H1), 7.64 (dd, 1H, *J* = 8.2 and 0.8 Hz, Carb-H8), 7.50 (ddd, 1H, *J* = 8.2, 7.2 and 1.2 Hz, Carb-H7), 7.26 (ddd, 1H, *J* = 8.2, 7.2 and 0.7 Hz, Carb-H6), 4.42 (t, 2H, *J* = 7.0 Hz, N-CH_2_), 1.83–1.73 (m, 2H, CH_2_), 1.35–1.12 (m, 10H, 5 × CH_2_), 0.81 (t, 3H, *J* = 7.1 Hz, CH_3_); HRMS (ESI, [M − H]^−^): found: 455.1816, requires for C_28_H_29_N_2_O_2_S: 455.1799.

*Methyl 2-Cyano-3-[5-(9-octylcarbazol-3-yl)thiophen-2-yl]acrylate* (**4B**). This compound was synthesized according to the esterification as outlined earlier. Yield: 99%; ^1^H-NMR (400 MHz, CDCl_3_) δ: 8.40 (d, 1H, *J* = 1.5 Hz, Carb-H4), 8.29 (s, 1H, vinylCN-H), 8.15 (ddd, 1H, *J* =8.0, 1.3 and 0.8 Hz Carb-H5), 7.78 (dd, 1H, *J* = 8.6 and 1.5 Hz, Carb-H2), 7.73 d (1H, *J* = 4.1 Hz, Th-H3), 7.50 (ddd, 1H, *J* = 8.3, 7.1 and 1.3 Hz, Carb-H7), 7.45 (d, 1H, *J* = 4.1 Hz, Th-H4), 7.43–7.37 (m, 2H, Carb-H1, H8), 7.28 (ddd, 1H, *J* = 8.0, 7.1, 1.1, 7.1 Hz, Carb-H6), 4.29 (t, 2H, *J* = 7.2 Hz, N-CH_2_), 3.91 (s, 3H, COOCH_3_), 1.93–1.82 (m, 2H, CH_2_), 1.46–1.18 (m, 10H, 5 × CH_2_), 0.86 (t, 3H, *J* = 7.1 Hz, CH_3_); ^13^C-NMR (100 MHz, CDCl_3_) δ: 163.8, 157.3, 146.9, 141.3, 141.1, 139.9, 133.7, 126.5, 124.5, 123.7, 123.5, 123.1, 122.7, 120.8, 119.7, 118.7, 116.4, 109.3, 109.2, 95.9, 53.1, 43.3, 31.8, 29.3, 29.2, 29.0, 27.3, 22.6, 14.1; HRMS (ESI, [M+H]^+^): found: 471.2121, requires for C_29_H_31_N_2_O_2_S: 471.2101.

### 3.2. Computation Methods

The compounds were modeled using Gaussian 09 D0.1 (Gaussian Inc, Wallingford, CT, USA) [61]. For all the calculations a 6-31G(d) basis set and a range of functionals, B3LYP [62] and CAM-B3LYP, were used. All calculations were carried out to Gaussians default criteria, with all optimized geometries showing no negative frequencies to ensure a minimum, rather than a saddle point, had been found. For solvent calculations the integral equation formalism Polarizable Continuum Model (IEF-PCM) [63,64] was used with Gaussian’s predefined definitions of the solvents. TD-DFT calculations were performed to the 30th state using the TD keyword in Gaussian to implement TD-DFT as defined within the Gaussian program [65], for solvent calculations the solvent molecule were not equilibrated. The calculations were performed using New Zealand eScience Infrastructure (NeSI, Auckand, New Zealand). The vibrational frequencies and orbitals were visualized using Molden [66] and GaussView 05W [67] (Gaussian Inc.) respectively. A scaling factor of between 0.94 and 0.97 was applied to the vibrational frequencies to account for anharmonicity, the values used are consistent with those determined in the literature [33,68]. The calculated normal Raman spectra were modelled for a 1064 nm excitation wavelength [53]. This uses the equation:

(3) $$\frac{\delta \sigma_{j}}{\delta \Omega }=\left(\frac{2^{4}\pi^{4}}{45}\right)\left(\frac{{\left(\upsilon_{0}-\upsilon_{j}\right)}^{4}}{1-\exp\left[\frac{-hc\upsilon_{j}}{kT}\right]}\right)\left(\frac{h}{8\pi^{2}c\upsilon_{j}}\right)S_{j}$$

where the Raman activity, *Sj* is provided from the Gaussian calculations, *ν*_0_ is the laser frequency, 1064 nm in this case, *ν_j_* is the frequency of the *j*th mode, $\frac{\delta \sigma_{j}}{\delta \Omega }$ is the observed different Raman cross-section, *h* and *c* are the Planck’s constant and speed of light respectively and $1-\exp\left[\frac{-hc\upsilon_{j}}{kT}\right]$ accounts for the population of *ν* = 0 of the *j*th state, with T being the temperature in Kelvin, in this case 298 K was used.

### 3.3. Physical Methods

For all measurements analytic grade solvents were used, as supplied. Unless otherwise stated all measurements were carried out at room temperature under standard atmospheric pressure. The experimental data was processed using a combination of Grams/AI 08 (ThermoFisher, Waltham, MA, USA) and OriginPro v9.0 (OriginLabs, Northampton, MA, USA).

Electronic absorbance spectra were recorded on either a Varian Cary 500 scan UV-vis-NIR spectrophotometer (Agilent, Santa Clara, CA, USA) equipped with the Cary WinUV software. A scan rate of 200 nm min^−1^ was employed between 300 and 800 nm (**1A**–**3A**) or a USB2000+UV-Vis-ES spectrometer (OceanOptics, Largo, FL, USA) and OceanView 1.5.0 software (OceanOptics) (**4A** and set B). Sample concentration varied from between 1 × 10^−4^ to 1 × 10^−6^ mol L^−1^. For acid, base and H_2_O treated spectra, an excess of NH_3_, HCl or distilled H_2_O was added.

FT-Raman spectra were measured using either a Bruker Equinox-55 FT-interferometer bench equipped with a FRA106/5 Raman accessory and utilizing OPUS 5.0 software (Bruker Optics, Billerica, MA, USA) (**1A**–**3A**) or Bruker MultiRAM (Bruker, Billerica, MA, USA) and OPUS 9.0 (Bruker) software. A Nd:YAG 1064 nm excitation laser was used and a liquid nitrogen cooled D418T Ge detector. The solid state samples were recorded with 2048 scans at 300 mW and 2 cm^−1^ resolution, while the solution samples were collected using 4096 scans at 400 mW and 4 cm^−1^ resolution.

Resonance Raman spectra were collected using a setup which has been previously described before [69]. In short, it is composed of an excitation beam and collection lens in a 135° backscattering arrangement. For set A a krypton ion laser (Innova 300C, Coherent Inc., Santa Clara, CA, USA) was used to provide excitation wavelengths (λ_exc_) of 350.7, 406.7 and 413.1 nm, a solid-state (CrystaLaser, Reno, NV, USA) was used for 448.0 nm and a Coherent Innova Sabre argon-ion laser for 457.9, 488.0 and 514.5 nm alongside an Acton SpectraPro500i spectrograph with Spec10 liquid-nitrogen-cooled CCD detector (Princeton Instruments, Trenton, NJ. USA). For set B and **4A** excitation at 350.7, 406.7 and 413.1 nm was made using a krypton ion laser, while crystal diode lasers were used for 375.0, 448.0 nm (CrystaLaser), 458.0, 491.0 and 515.0 nm (Cobolt, Solna, Sweden) excitation alongside an Isoplane SCT320 spectrometer with PyLon CCD (Princeton Instruments, Trenton, NJ. USA) cooled to −120 °C with liquid nitrogen. Notch filters (Kaiser Optical, Inc., Ann Arbor, MI, USA) or long-pass filters (Semrock, Inc., Rochester, NY, USA) matched to these wavelengths were used to remove the laser excitation line. Winspec (v2.5.8.1) software (Roper Scientific, Princeton Instruments) was used to control the CCD. The resonance Raman spectra were recorded at 1 × 10^−4^ mol L^−1^.

Emission spectra were collected using a diode laser, [448.0 nm (CrystaLaser) or 355 nm (Cobolt)] for excitation and a SP2150 (Acton, Princeton Instruments) spectrometer with an air cooled CCD (Pixis 100) on a 90° bench top setup. Winspec (v2.5.8.1) software (Roper Scientific, Princeton Instruments) was used to collect the spectra.

For the **1A**–**3A** emission spectra were collected on the same setup as discussed for resonance Raman, using the Innova I-302 krypton ion laser at 350.7 nm for excitation. While for the rest a diode laser, [448.0 nm (CrystaLaser)] for excitation and a SP2150 (Acton, Princeton Instruments) spectrometer with an air cooled CCD (Pixis 100, Princeton Instruments, Trenton, NJ. USA)) on a 90° bench top setup. A Quantum NorthWest TC125 temperature controller (Liberty Lake, WA, USA) was used to vary temperature for the ranges of 248 K to 293 K across all solvents. Sample concentration was typically 1 × 10^−5^ mol L^−1^.

Quantum yields were measured using a FS5 (Edinburgh Instruments, Livingston, UK) with integrating sphere module. 325.4 nm light from a xenon arc lamp as used for illumination.

Lifetimes were recorded using TCSPC photon counting on a FS5 (Edinburgh Instruments) with a EPLED-320 diode laser (Edinburgh Instruments, UK), emitting at 325.4 nm for excitation.

## 4. Conclusions

The synthesis, optical characterization and computational modelling of eight dyes based on 3-{5-[2-(9-octylcarbazol-3-yl)ethenyl]thiophen-2-yl}-2-cyanoacrylic acid framework (**1A**) are reported. A broad absorption band is observed between 410 and 520 nm, and shown to be charge transfer in nature from the carbazole group to cyano-carboxylate using resonance Raman spectroscopy, emission spectroscopy and DFT calculations. The energy and intensity of this transition is modulated by the elongation of the thiophene linker. It is observed that the bithiophene linker appears to have a much greater effect on optical properties than the terthiophene. The emission quantum yield and lifetimes are greatest for the bithiophene system and it appears that this is the optimal length to facilitate the donor-acceptor interaction. The bithiophene linker has a non-radiative rate constant an order of magnitude less than the other systems. It was also determined that the inclusion of the ethenyl linker between donor and bridge resulted in a blue shift of the absorbance spectrum and slight decrease in the degree of charge moved during the excitation. Significant solvatochromisim was observed in both absorption and emission data, which was attributed to contributions from deprotonation of the cyano-carboxylate and solvent stabilization. Modest changes in dipoles were calculated for all compounds using Lippert Mataga analysis.

## Figures and Tables

**Table 1:** Lowest energy transition (λ_max_) for the compound studied, in DCM, as measured experimentally and calculated using TD-DFT.

Compound	Experimental		Calculated					
			B3LYP			CAM-B3LYP		
	nm	eV	nm	eV	eV_expt_–eV_b3lyp_	nm	eV	eV_expt_–eV_cam-b3lyp_
**1A**	487	2.55	533	2.33	0.22	452	2.74	−0.20
**2A**	509	2.44	607	2.04	0.39	487	2.55	−0.11
**3A**	508	2.44	664	1.87	0.57	508	2.44	0.00
**4A**	467	2.66	480	2.58	0.07	385	3.22	−0.57
**1B**	494	2.51	522	2.38	0.13	422	2.94	−0.43
**2B**	517	2.40	596	2.08	0.32	452	2.74	−0.34
**3B**	519	2.39	656	1.89	0.50	508	2.44	−0.05
**4B**	473	2.62	491	2.53	0.10	392	3.16	−0.54

**Table 2:** Calculated values for the dihedral between carbazole and thiophene units for structures calculated at B3LYP level, for **1A**/**1B** and **4A**/**4B**, as assigned in Figure 4.

Compound	Dihedral Angle/°
1A	0.4
1B	0.1
4A	23.8
4B	24.3

**Table 3:** The emission maximum observed for the compounds studied in a range of solvents.

Compound	λ_em_/nm				
	Toluene	CHCl_3_	DCM	DMF	MeCN
**1A**	577	641	642	561	659
**2A**	605	652	703	586	654
**3A**	622	672	715	602	649
**4A**	527	588	577	518	517
**1B**	590	617	635	665	662
**2B**	597	663	684	716	731
**3B**	620	701	718	627	611
**4B**	522	552	567	597	596

**Table 4:** Excited state lifetimes, as measure by photon counting, and quantum yields for the set B compounds, in toluene and DCM. Alongside calculated radiative and non-radiative decay rates.

Compound	Lifetime/ns		Quantum Yield		Decay Rates in DCM/s^−1^	
	Toluene	DCM	Toluene	DCM	*k*_r_	*k*_nr_
1B	*	5.1 ± 0.4	0.07	0.35	6.9 × 10^7^	1.3 × 10^8^
2B	5.5 ± 0.1	17.9 ± 0.4	0.46	0.70	3.9 × 10^7^	1.7 × 10^7^
3B	*	6.1 ± 0.2	0.23	0.23	3.8 × 10^7^	1.3 × 10^8^
4B (450 nm)	5.8 ± 0.1	2.5 ± 0.1	0.02	0.05	1.4 × 10^7 #^	2.7 × 10^8 #^
4B (550 nm)	6.4 ± 0.1	3.5 ± 0.2				

* Lifetime too short to be clearly distinguished from the instrument response; ^#^ Lifetime for peak at 550 nm was used.

## Supplementary Materials

The following are available online, Tables S1–S3; Figures S1–S14.
