# Supplementary material for: Modulation of Donor-Acceptor Distance in a Series of Carbazole Push-Pull Dyes; A Spectroscopic and Computational Study
Source: Molecules. 2018 Feb 14;23(2):421. doi: 10.3390/molecules23020421 (PMC6017769; doi:10.3390/molecules23020421)
Supplement: Supplementary file 1 [file molecules-23-00421-s001.pdf]

Modulation of Donor-Acceptor Distance in a Series of Carbazole Push-Pull Dyes; A Spectroscopic and Computational Study

Joshua J. Sutton [1], Jonathan E. Barnsley [1], Joseph I. Mapley [1], Pawel Wagner [2,3,], David L. Officer [2,3,*], Keith C. Gordon [1,*]*

[1] MacDiarmid Institute for Advanced Materials and Nanotechnology, Department of Chemistry, University of Otago, P. O. Box 56, Dunedin, New Zealand

[2] ARC Centre of Excellence for Electromaterials Science, University of Wollongong, NSW Australia

[3] Intelligent Polymer Research Institute/AIIM Faculty, Innovation Campus, University of Wollongong, NSW Australia

* Author to whom correspondence should be addressed (e-mail keith.gordon@otago.ac.nz)

Table S1. Optimised structures and energy for various thiophene configurations in **2A**/**2B** and **3A**/**3B**, as modeled at B3LYP/6-31G(d) level.

		Energy / a.u.	Relative Energy / a.u.	Relative Energy / kJ mol^{-1}
3A	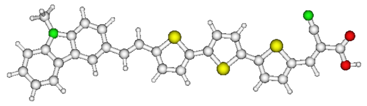	-2647.8493	0	0
	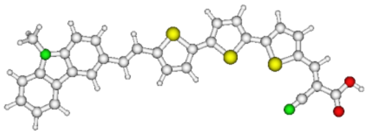	-2647.8474	0.001858	4.88
	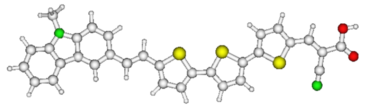	-2647.8478	0.001501	3.94
	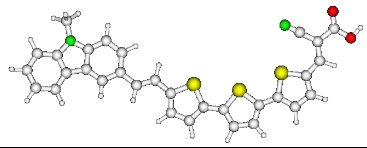	-2647.8456	0.003714	9.75
2A	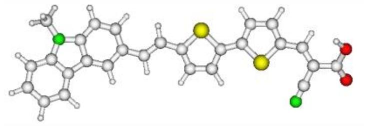	-2096.0208	0	0
	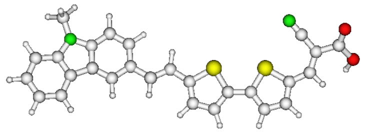	-2096.0190	0.001819	4.77
3B	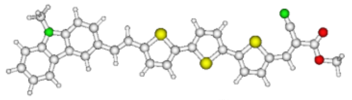	-2687.1560	0	0
	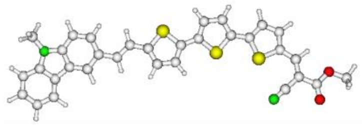	-2687.1544	0.001658	4.35
	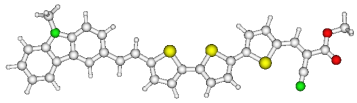	-2687.1548	0.001236	3.24
	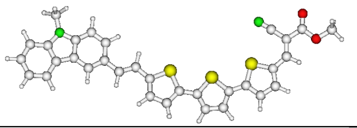	-2687.1529	0.003164	8.31
2B	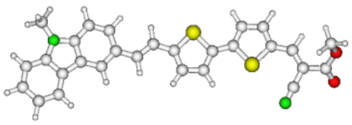	-2135.3208	0	0
	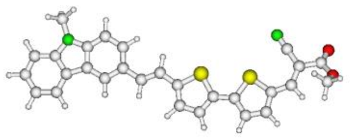	-2135.3192	0.001642	4.31

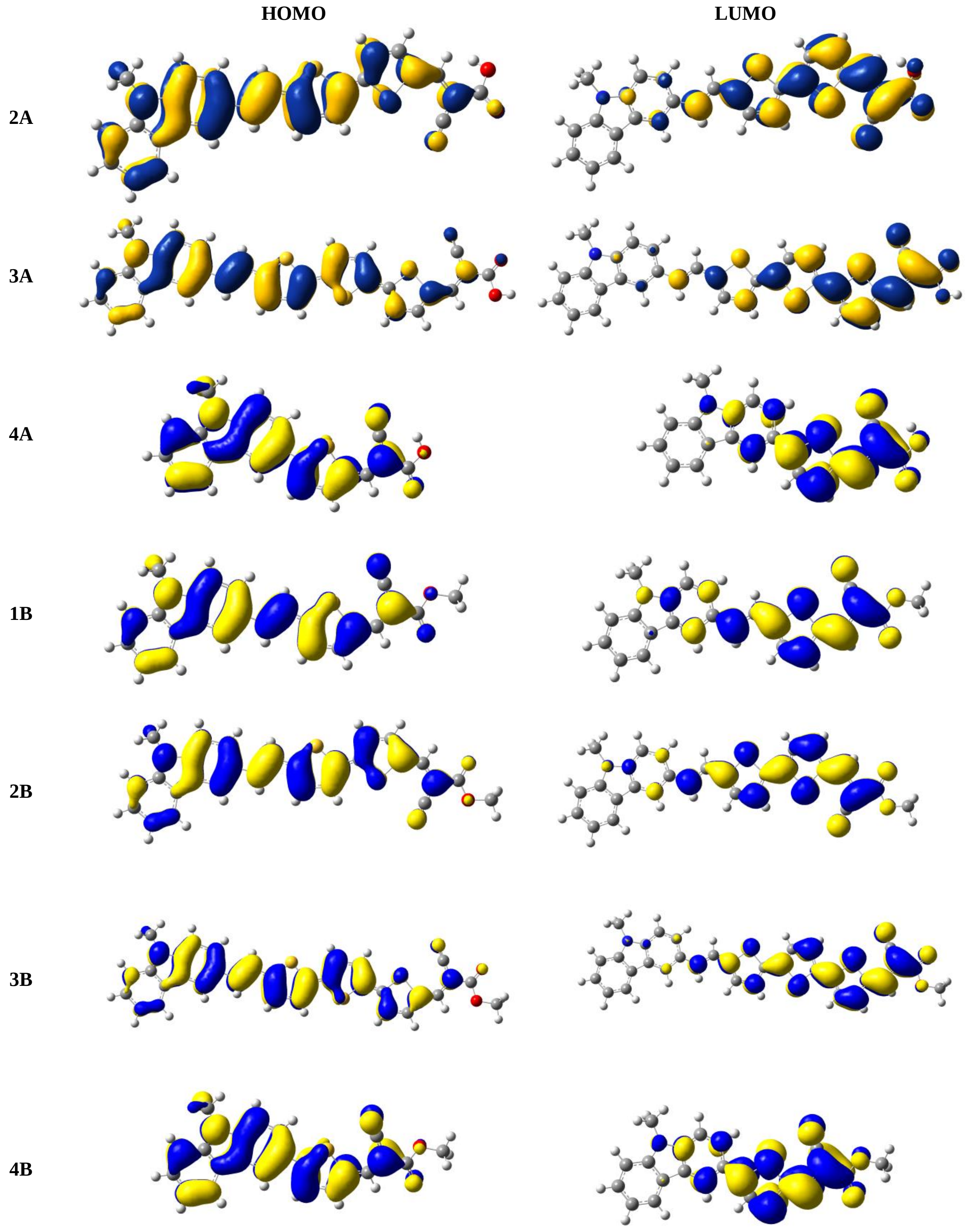

Figure S1: B3LYP calculated HOMO and LUMO orbitals for **2A-4A** and **1B-4B**.

Table S2. Orbital population density for the compound series, calculated by B3LYP and CAM-B3LYP in DCM. HOMO to LUMO donation was predicted at 99% for B3LYP and 82% for CAM-B3LYP.

		**B3LYP Orbital Population Density**			**CAM-B3LYP Orbital Population Density**		
		**carbazole**	**thiophene**	**cyanoacrylate**	**carbazole**	**thiophene**	**cyanoacrylate**
	HOMO	57	33	10	57	35	8
**1A**	LUMO	10	47	34	8	49	43
	Δ	-47	14	33	-49	14	35
	HOMO	43	51	6	42	53	5
**2A**	LUMO	4	55	41	3	57	41
	Δ	-39	4	35	-39	4	36
	HOMO	33	63	4	32	66	3
**3A**	LUMO	2	60	38	1	62	37
	Δ	-31	-3	34	-31	-4	34
	HOMO	74	17	10	70	20	9
**4A**	LUMO	11	38	51	9	39	52
	Δ	-63	21	41	-61	19	43
	HOMO	54	35	11	54	37	9
**1B**	LUMO	10	48	42	8	49	44
	Δ	-44	13	31	-46	12	33
	HOMO	35	57	8	48	47	4
**2B**	LUMO	5	58	37	3	56	40
	Δ	-30	1	29	-45	9	36
	HOMO	32	63	4	35	61	4
**3B**	LUMO	2	62	36	2	63	35
	Δ	-30	-1	32	-33	2	31
	HOMO	72	18	10	67	22	10
**4B**	LUMO	11	38	51	9	39	52
	Δ	-61	20	41	-58	17	42

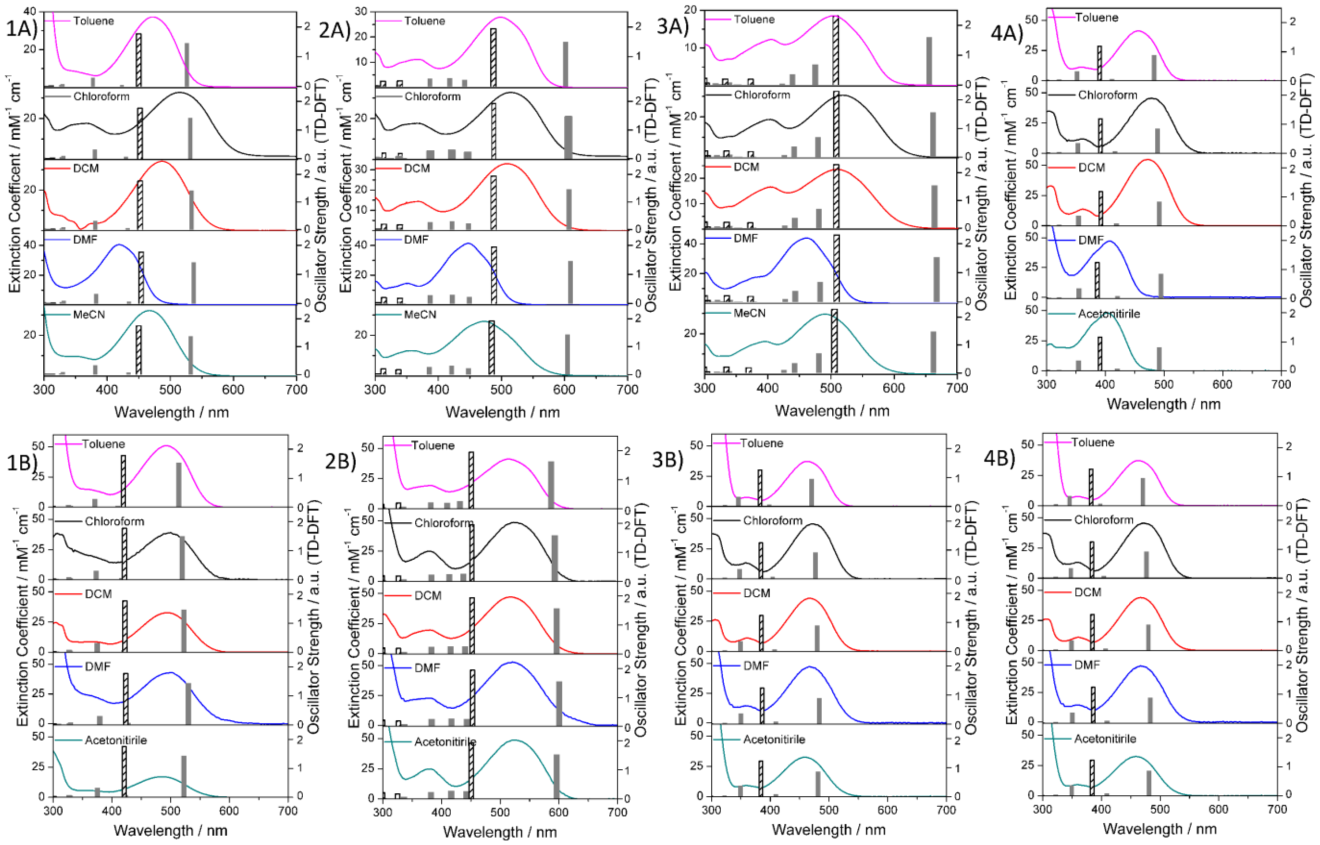

Figure S2: Experimentally collected electronic absorbance data for all compounds, as labeled, in a variety of solvents, showing DFT calculated transitions with B3LYP represented in grey and CAM-B3LYP represented in black dash.

Table S3. Lowest energy transition for the compounds studied in a range of solvents, as measured experimentally and calculated using TD-DFT

		EXPT		B3LYP			CAM-B3LYP		
	Solvent	nm	eV	nm	eV	eVexpt-eVb3lyp	nm	eV	eVexpt-eVcam-b3lyp
1A	Toluene	473	2.62	526	2.36	0.26	450	2.76	-0.13
	Chloroform	491	2.53	531	2.34	0.19	451	2.75	-0.22
	DMF	419	2.96	536	2.31	0.65	453	2.74	0.22
	MeCN	468	2.65	532	2.33	0.32	449	2.76	-0.11
2A	Toluene	499	2.48	601	2.06	0.42	487	2.55	-0.06
	Chloroform	514	2.41	606	2.05	0.37	487	2.55	-0.13
	DMF	447	2.77	609	2.04	0.74	488	2.54	0.23
	MeCN	473	2.62	605	2.05	0.57	484	2.56	0.06
3A	Toluene	506	2.45	656	1.89	0.56	508	2.44	0.01
	Chloroform	519	2.39	662	1.87	0.52	508	2.44	-0.05
	DMF	461	2.69	667	1.86	0.83	509	2.44	0.25
	MeCN	491	2.53	663	1.87	0.66	505	2.46	0.07
4A	Toluene	456	2.72	483	2.57	0.15	390	3.18	-0.46
	Chloroform	477	2.60	489	2.54	0.06	391	3.17	-0.57
	DMF	407	3.05	494	2.51	0.54	386	3.21	-0.17
	MeCN	404	3.07	492	2.52	0.55	391	3.17	-0.10
1B	Toluene	490	2.53	513	2.42	0.11	420	2.95	-0.42
	Chloroform	500	2.48	519	2.39	0.09	421	2.95	-0.47
	DMF	502	2.47	530	2.34	0.13	423	2.93	-0.46
	MeCN	485	2.56	522	2.38	0.18	420	2.95	-0.40
2B	Toluene	511	2.43	587	2.11	0.31	451	2.75	-0.32
	Chloroform	522	2.38	593	2.09	0.28	452	2.74	-0.37
	DMF	517	2.40	600	2.07	0.33	453	2.74	-0.34
	MeCN	505	2.46	596	2.08	0.37	451	2.75	-0.29
3B	Toluene	519	2.39	646	1.92	0.47	508	2.44	-0.05
	Chloroform	523	2.37	653	1.90	0.47	508	2.44	-0.07
	DMF	514	2.41	659	1.88	0.53	509	2.44	-0.02
	MeCN	507	2.45	655	1.89	0.55	506	2.45	0.00
4B	Toluene	456	2.72	483	2.57	0.15	390	3.18	-0.46
	Chloroform	477	2.60	489	2.54	0.06	391	3.17	-0.57
	DMF	407	3.05	494	2.51	0.54	386	3.21	-0.17
	MeCN	404	3.07	492	2.52	0.55	391	3.17	-0.10

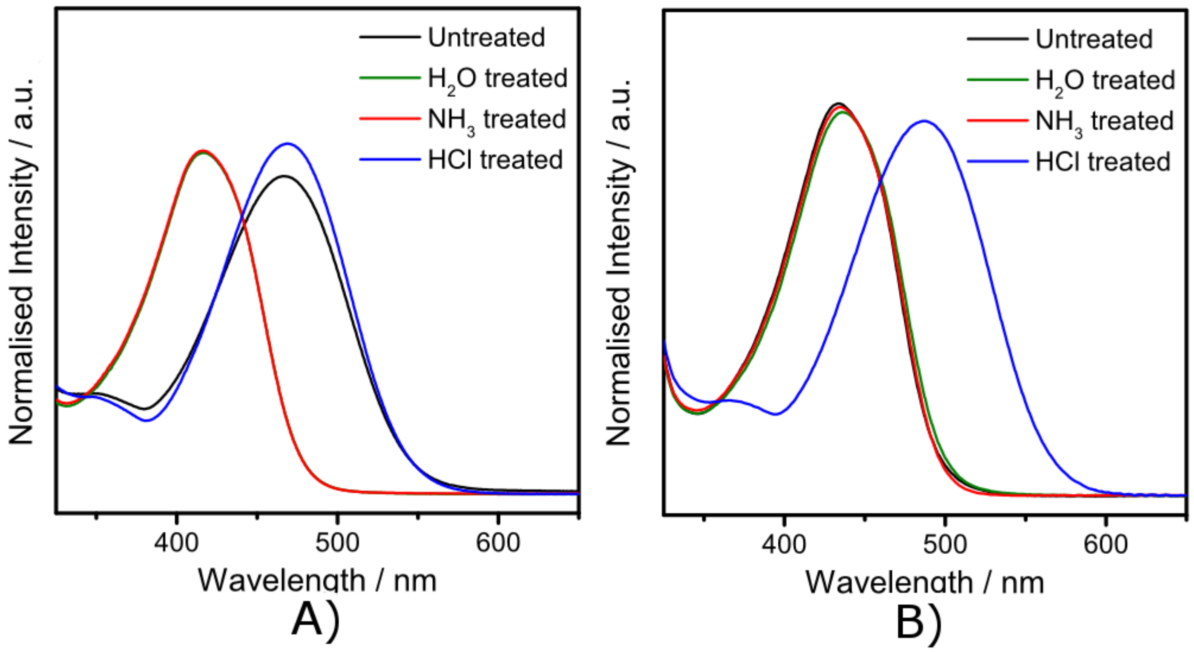

Figure S3: Electronic absorbance of **1A** measured in (A) MeCN and (B) DMF with acid and base treatment.

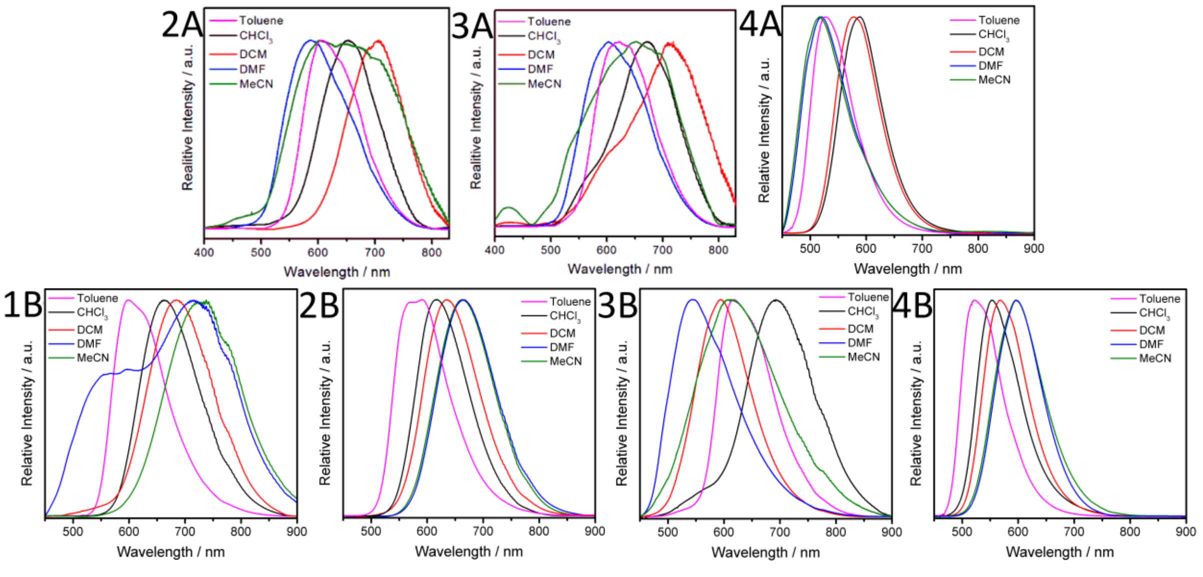

Figure S4: Emission data for **2A-4A** and **1B-4B** recorded in a number of solvents.

Table S4. Change in dipole (Δμ) upon excitation, as calculated from Lippert-Mataga analysis of experimental absorbance and emission data.

	Δμ
**1A**	11.2
**2A**	13.1
**4A**	11.0
**1B**	12.0
**2B**	15.2
**4B**	13.6

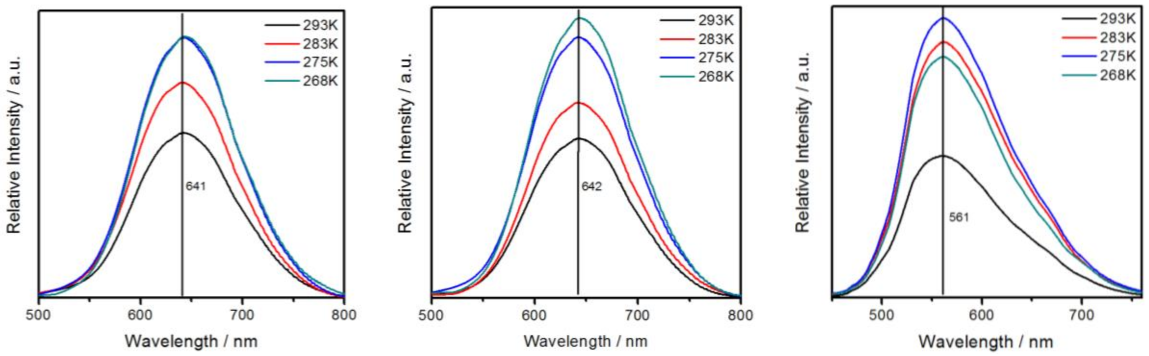

a) b) c)

Figure S5: Variable temperature emission of **1A** measured in a) chloroform, b) dichloromethane and c) DMF.

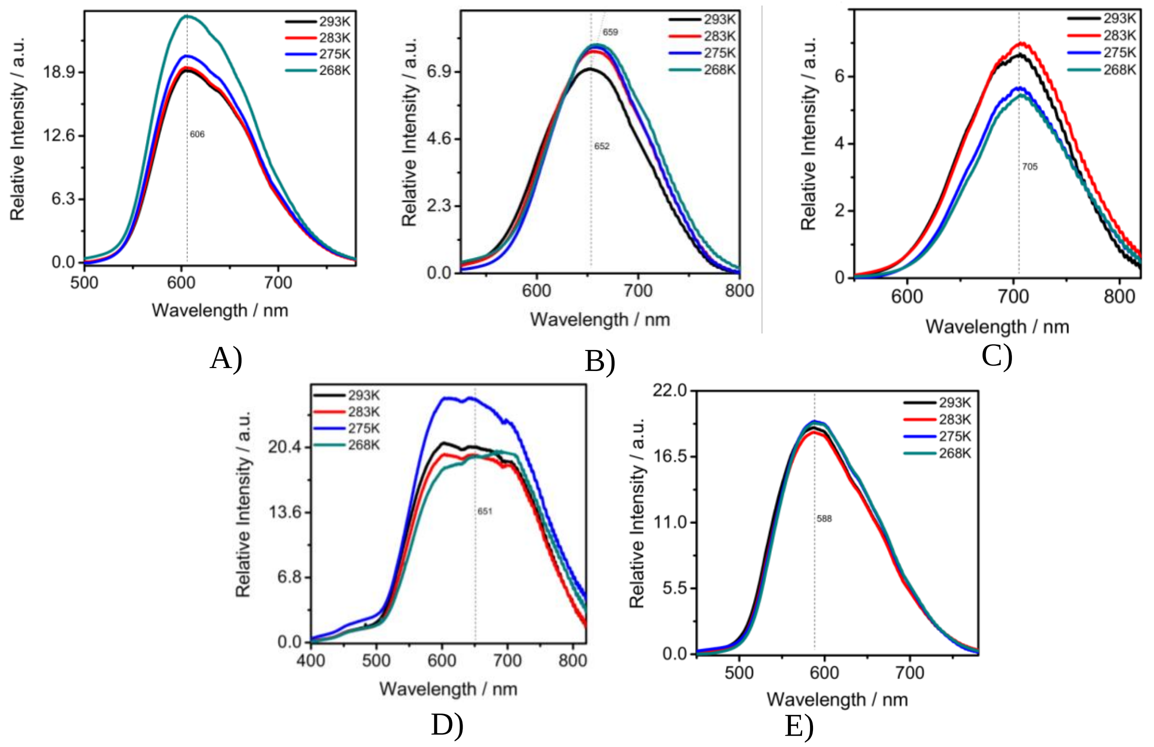

Figure S6: Variable temperature emission of **2A** measured in a) toluene, b) chloroform, c) dichloromethane, d) acetonitrile and e) dimethylformamide.

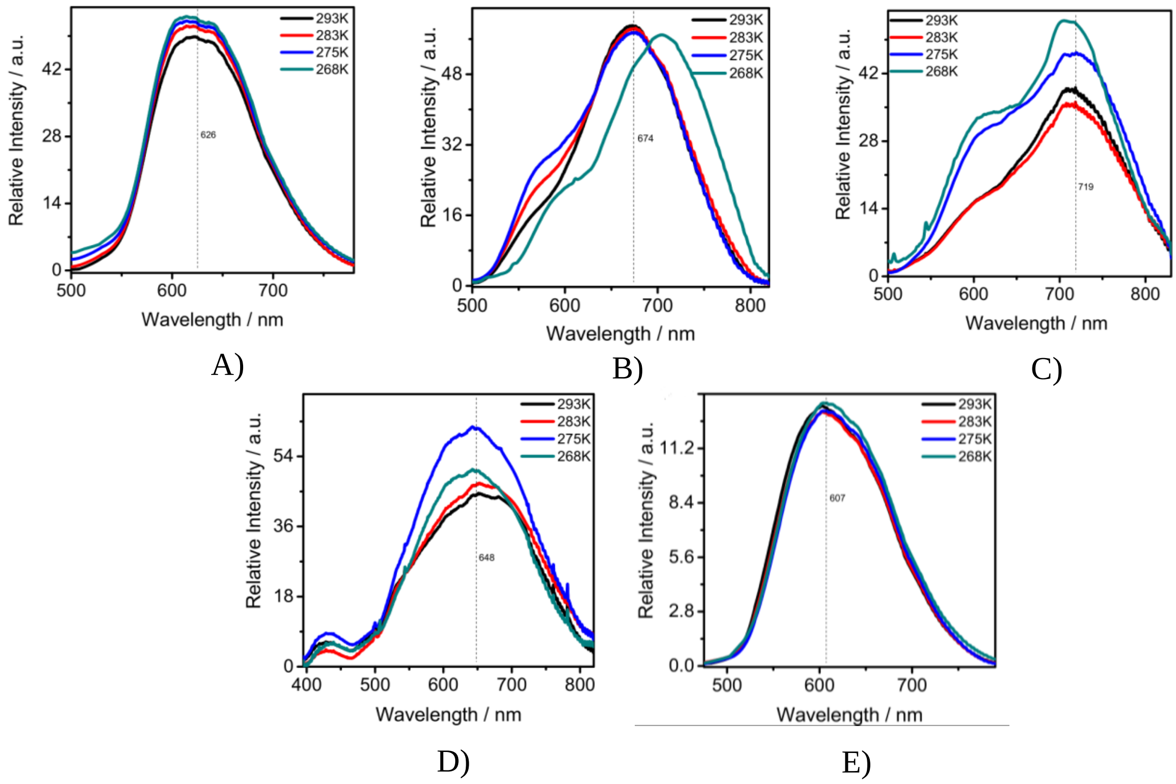

Figure S7: Variable temperature emission of **3A** measured in a) toluene, b) chloroform, c) dichloromethane, d) acetonitrile and e) dimethylformamide

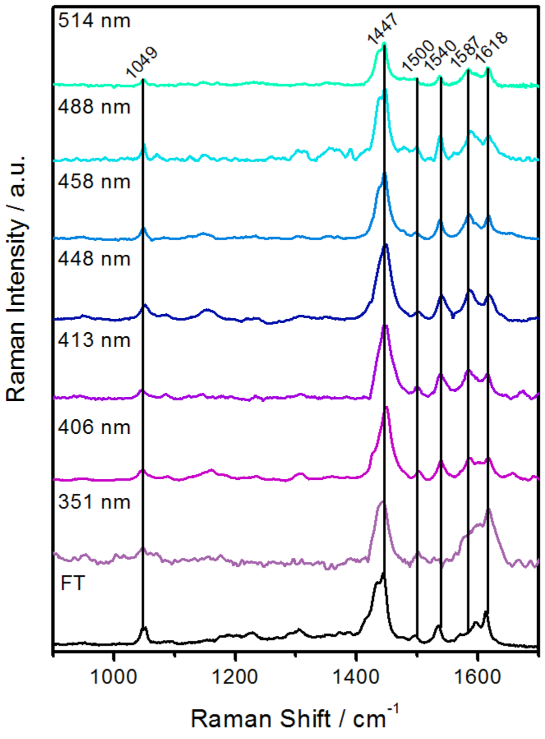

A)

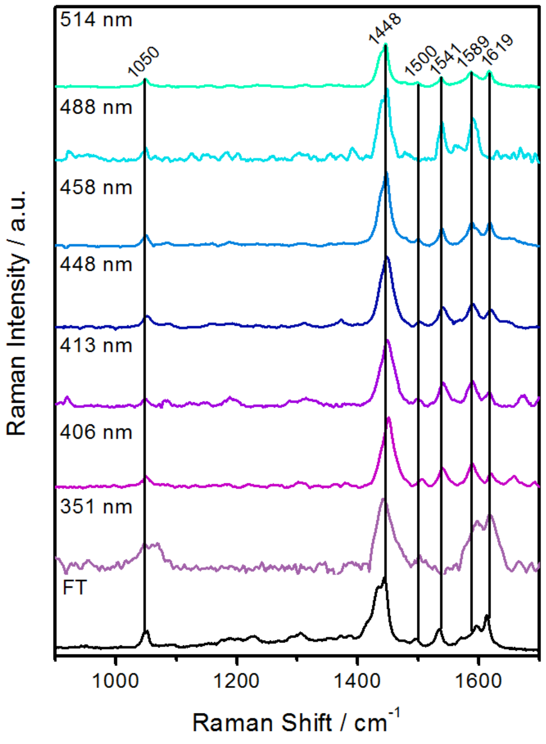

B)

Figure S8: Measure resonance Raman spectroscopy of **2A** in a) DCM and b) acetonitrile.

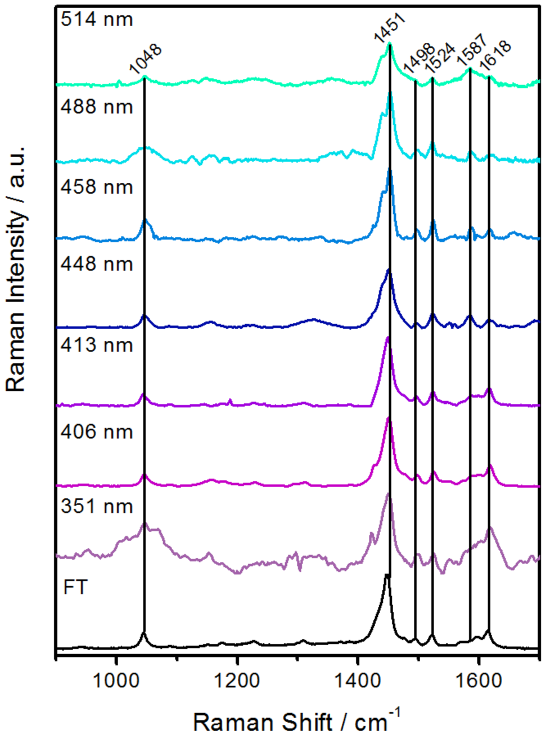

A)

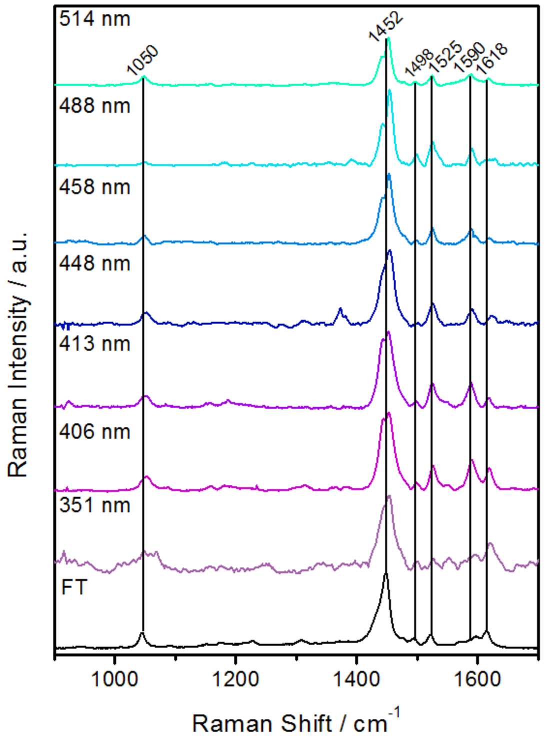

B)

Figure S9. Measure resonance Raman spectroscopy of **3A** in a) DCM and b) acetonitrile.

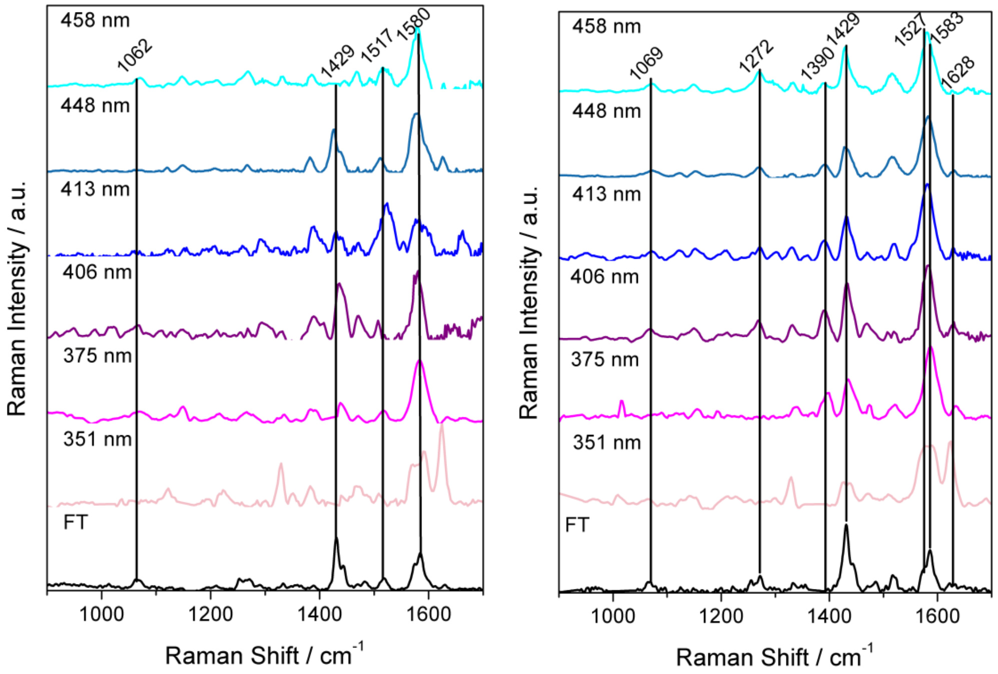

A) B)

Figure S10. Measure resonance Raman spectroscopy of **4A** in a) DCM and b) acetonitrile.

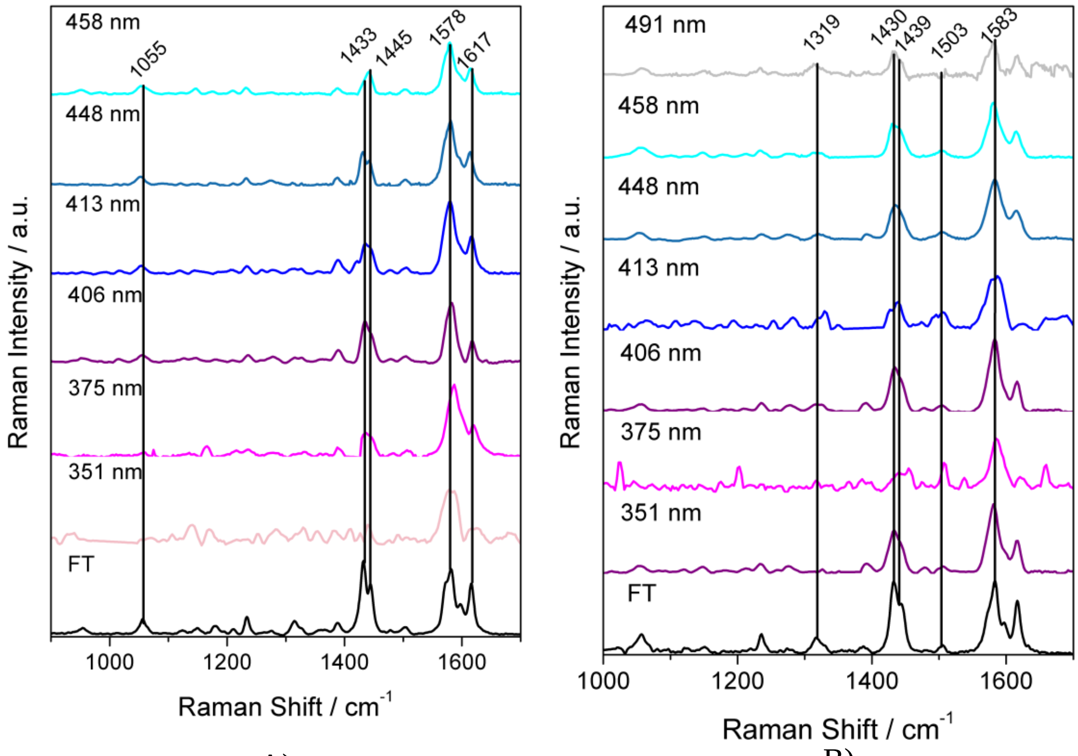

A) B)

Figure S11. Measure resonance Raman spectroscopy of **1B** in a) DCM and b) acetonitrile.

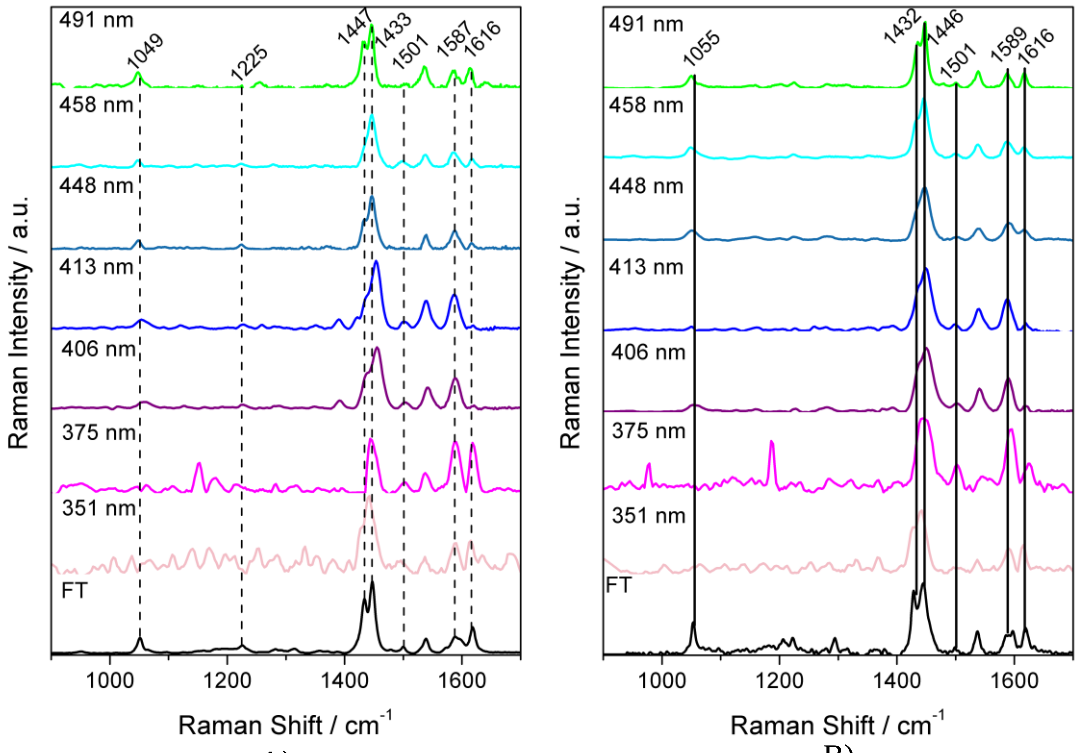

A) B)

Figure S12. Measure resonance Raman spectroscopy of **2B** in a) DCM and b) acetonitrile.

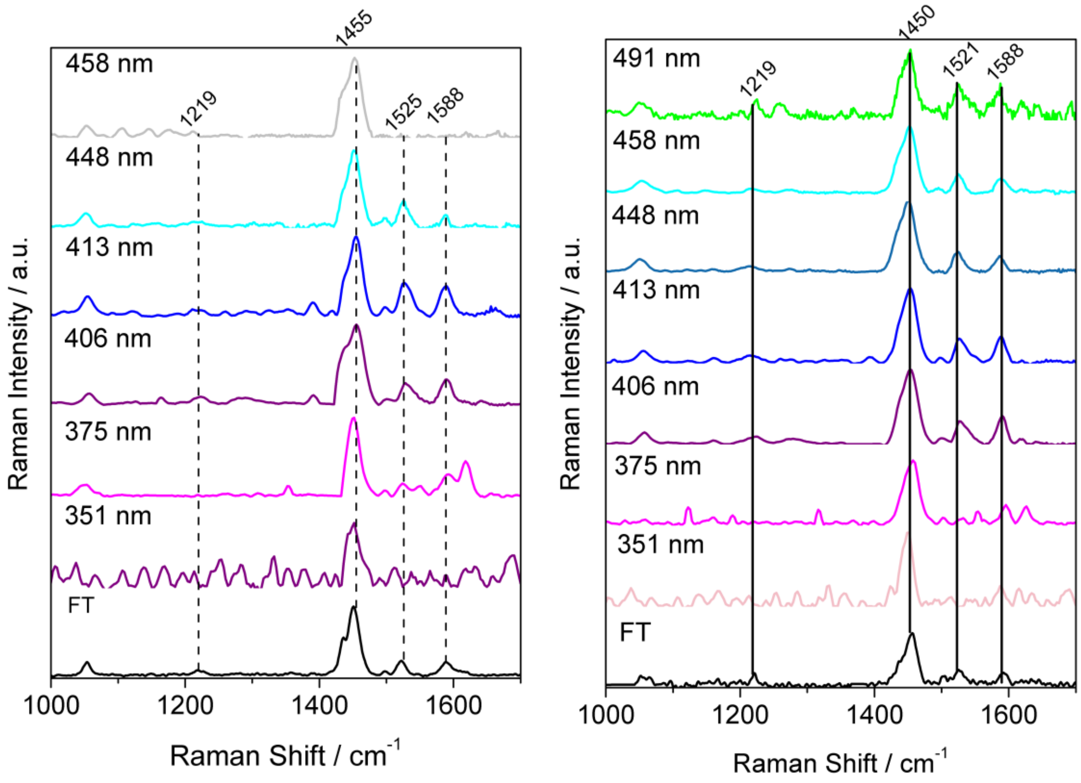

A) B)

Figure S13. Measure resonance Raman spectroscopy of **3B** in a) DCM and b) acetonitrile.

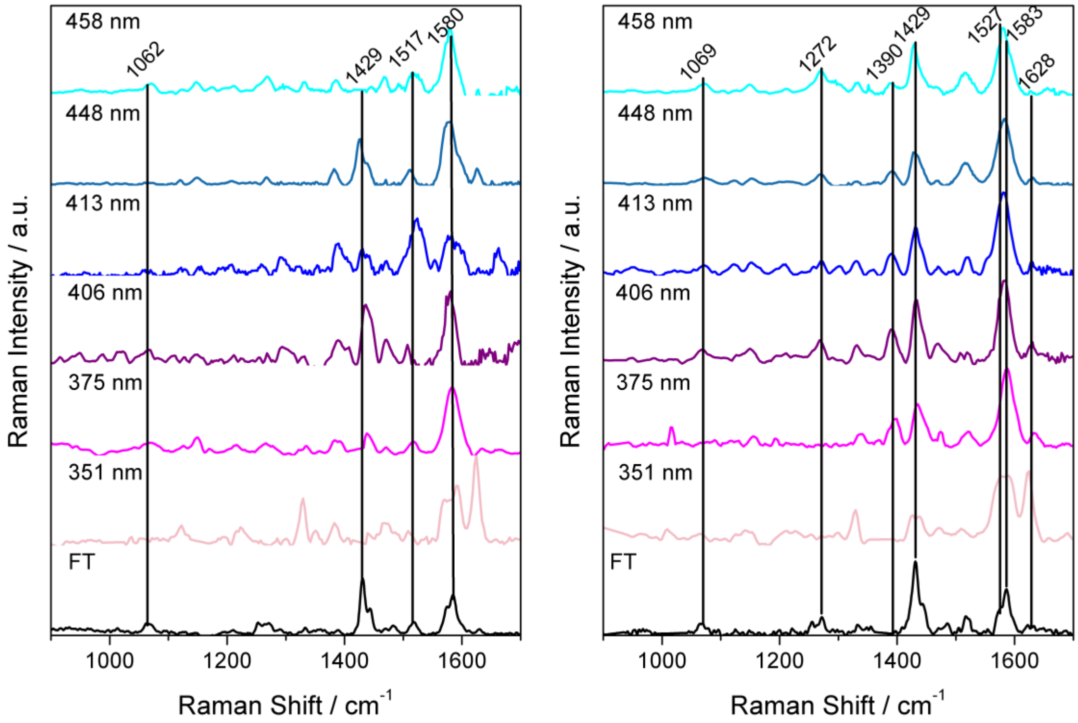

A) B)

Figure S14. Measure resonance Raman spectroscopy of **4B** in a) DCM and b) acetonitrile.

$$\phi = \frac{k_r}{k_r + k_{nr}} = k_r \tau \qquad \text{(S1)}$$

Where φ is the quantum yield, k_r is the radiative decay rate, k_{nr} is the non-radiative decay rate and τ is the lifetime (s).
